# Structure, characterisation and application of an unspecific peroxygenase from *Daldinia childiae*

**DOI:** 10.1039/d6cb00141f

**Published:** 2026-06-11

**Authors:** Alexander McKenzie, Claudia Clark, Katy A. S. Cornish, Jiacheng Li, Jack Domenech, Benjamin Melling, Miles P. H. Ralston, Jared Cartwright, Nicholas P. Mulholland, William P. Unsworth, Gideon Grogan

**Affiliations:** a Department of Chemistry, University of York Heslington York YO10 5DD UK william.unsworth@york.ac.uk gideon.grogan@york.ac.uk; b Department of Biology, University of York Heslington York YO10 5DD UK; c Syngenta, Jealott's Hill International Research Centre Bracknell Berkshire RG42 6EY UK

## Abstract

Unspecific peroxygenases (UPOs) have emerged as useful biocatalysts for the scalable and selective oxygenation of a large variety of organic molecules. UPOs have been divided into family I and family II enzymes, dependent upon sequence similarity and molecular weight, with family I being shorter in sequence. Here we report the characterisation and application of the family I UPO from *Daldinia childiae* (*Dch*UPO). The enzyme was expressed in both *Escherichia coli* and *Komagataella phaffii*, yielding protein for kinetic and structural studies and biocatalytic application respectively. The structure of the enzyme revealed notable differences in the active site tunnel, compared with the well-studied family I artUPO, including F79 for V69 and F171 for I160. Notably, these differences were manifested in selectivity divergent from other UPOs when *Dch*UPO was applied to preparative biotransformations; for example, (–)-menthol was converted exclusively into *cis*-6-hydroxymenthol in contrast to artUPO, which gave exclusively the tertiary alcohol 2,8-menthanediol.

## Abstract 2

The characterisation of the family I UPO from *Daldinia childiae* (*Dch*UPO) is described, in addition to its application in small molecule oxygenation reactions.

## Graphical abstract

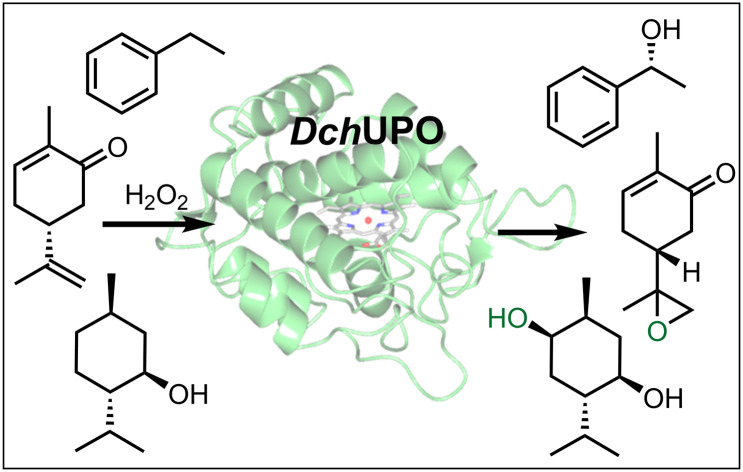

## Introduction

**Scheme 1:**
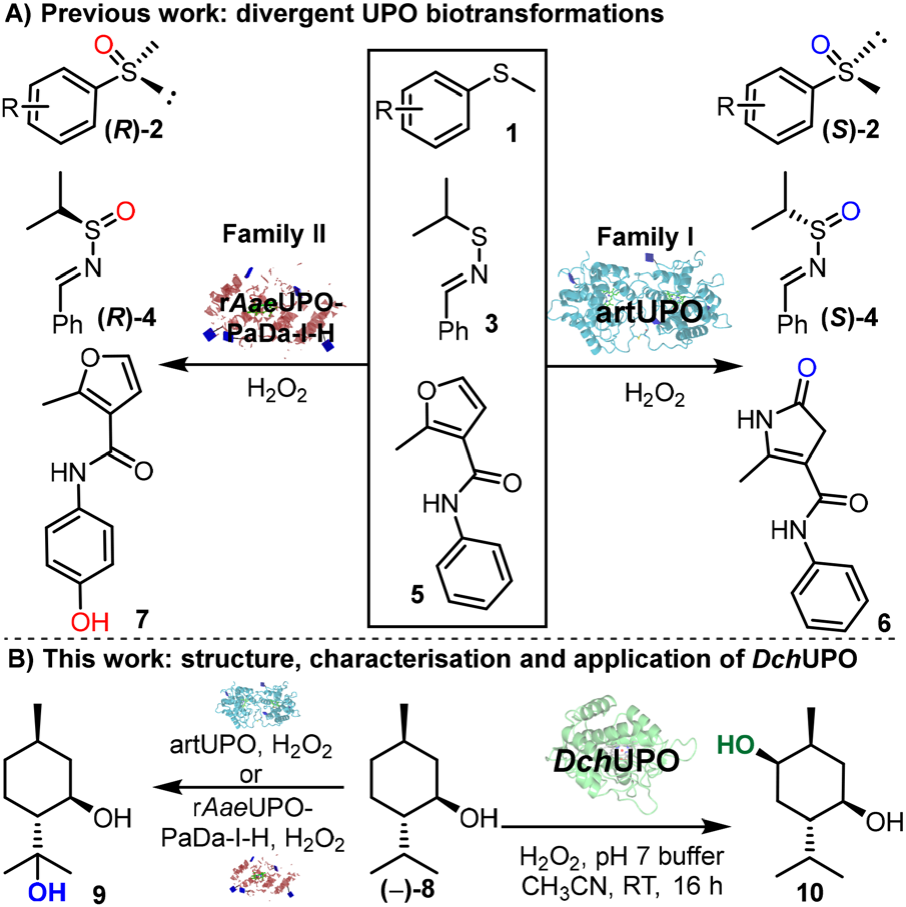
(A) Previous work: Divergent UPO biotransformations promoted by family I and family II unspecific peroxygenases; (B) this work: structure, characterisation and application to the selective oxygenation of menthol.

**Fig. 1:**
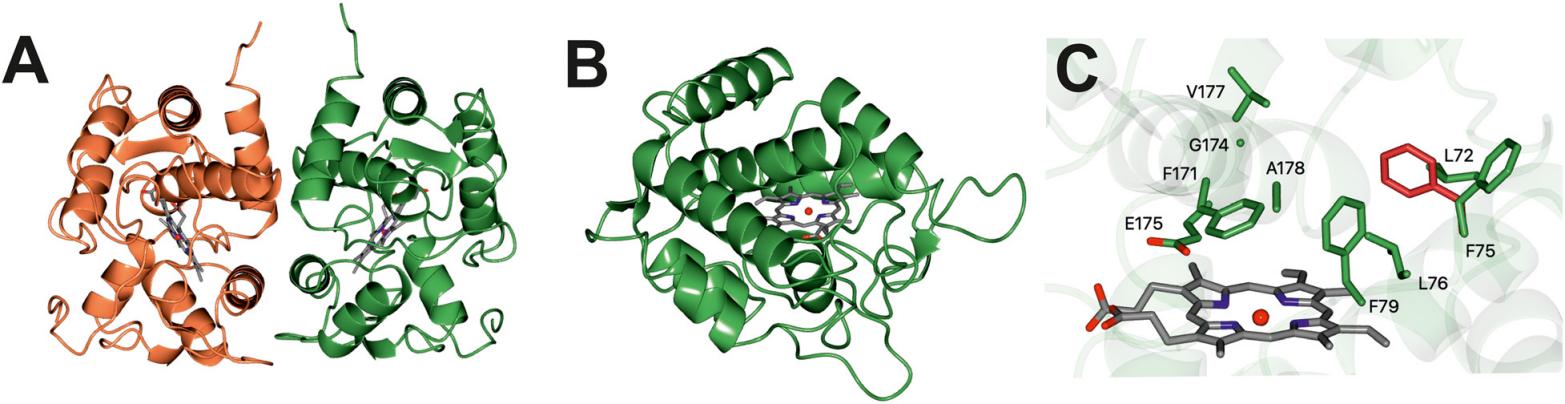
(A) Two molecules of the *Dch*UPO monomer in the asymmetric unit obtained using X-ray crystallography; (B) monomer B of *Dch*UPO; (C) active site of *Dch*UPO with amino acid side chains in the active site and approach tunnel labelled. F75 is shown in two conformations: those observed in monomer B and A are shown in green and coral respectively.

The selective oxygenation of hydrocarbons is an ongoing challenge in synthetic chemistry as abiotic oxidation methods are often unselective and require toxic reagents and/or harsh reaction conditions. Consequently, biocatalytic approaches to hydroxylation have been studied intensively and have for the most part focused on the activity of cytochromes P450 (P450s);^1–3^ heme-dependent oxygenases that use molecular oxygen, an external reductant (typically NAD(P)H) and auxiliary electron transfer proteins to form the active oxidant ‘Compound I’, an iron(iv) oxo species, in the active site.^4^ The requirements for reagents additional to the heme binding domain for P450 catalysis render their application for *in vitro* biocatalysis problematic. The discovery of unspecific peroxygenases (UPOs), which are single-domain heme-dependent oxygenases secreted by fungi, by Hofrichter and co-workers in 2004,^5^ identified a new group of useful oxygenative biocatalysts with a similar reactivity profile to that of P450s. A crucial difference between P450s and UPOs is that the addition of only hydrogen peroxide to a single protein domain is required to promote the formation of Compound I in UPOs to catalyse the oxygenation reaction.^6^ This greatly simplifies *in vitro* biocatalytic applications, especially as UPOs are stable, easy to express at a large volume, and amenable to storage as lyophilised powders for long periods. Since the initial discovery of the prototypical enzyme from *Agrocybe aegerita* (*Aae*UPO),^5^ many different enzymes from different fungal organisms have been described and applied to the transformation of organic substrates.^7–9^ These investigations have been greatly assisted by the development of robust methods for their heterologous expression in easy-to-grow hosts such as *Komagataella phaffii* (formerly *Pichia pastoris*)^10–13^ and in some cases *Escherichia coli*.^14–16^ The description of many UPOs has permitted their subdivision into two broad families based on their amino acid sequence length:^17^ family I enzymes, typified by the enzyme from *Marasmius rotula* (*Mro*UPO),^18^ have a mean molecular weight of ≈26 kDa; family II enzymes such as *Aae*UPO^5^ are longer, with a mean molecular weight of ≈44 kDa, by virtue of an extended C-terminal domain. Interestingly, family I and II UPOs can exhibit divergent behaviour with respect to their oxygenation reactions.^16,19–21^ For example, in previous work we have shown that the family I enzyme artUPO (‘artificial peroxygenase’)^16,22^ transforms sulfides 1 into (*S*)-sulfoxide products (*S*)-2, while the PaDa-I variant of the family II enzyme *Aae*UPO^10,11^ (‘r*Aae*UPO-PaDa-I-H’ in this case; the ‘H’ indicates that the construct features a C-terminal histidine tag)^23^ produces (*R*)-sulfoxide products (*R*)-2 (Scheme 1A).^16^ These differences may be attributed to differences in their active sites: family II enzymes, such as r*Aae*UPO-PaDa-I,^24^ feature a number of phenylalanine residues in this region, perhaps conferring superior selectivity; however the active sites of family I enzymes such as *Mro*UPO^25^ are not so sterically restricted and may overall be better at transforming larger substrates (Fig. 1).

More recently, we uncovered similar enantio-divergent reactivity for the biotransformations of sulfenimines (*e.g.*3), with (*S*)- and (*R*)-sulfinimines (*S*)-4 and (*R*)-4 generated in high ee from artUPO and r*Aae*UPO-PaDa-I-H respectively (Scheme 1A).^21^ The same pair of UPOs can also enable biotransformations with divergent chemoselectivity; for example, artUPO promotes the biotransformation of the agrochemical fenfuram 5 into pyrollinone 6 (in the presence of ammonium ions in the crude enzyme secretate), whereas under the same conditions r*Aae*UPO-PaDa-I-H catalyses aromatic hydroxylation to give 7 (Scheme 1A).^20^

In this manuscript, we describe work to extend our research into the activities and reactions of family I UPOs to the unspecific peroxygenase from *Daldinia childiae* (*Dch*UPO). Expression of *Dch*UPO was achieved first in *E. coli*, affording purified enzyme that permitted its analysis using kinetics and X-ray crystallography. Expression was then also performed using *Komagataella phaffii*, enabling the production of substantial amounts of *Dch*UPO for application in the preparative scale oxygenation of terpenes. Most notably, *Dch*UPO enabled the regio- and stereo-selective transformation of (–)-menthol (–)-8 into *cis*-6-hydroxymenthol 10 (Scheme 1B), in contrast to the outcome of biotransformations using artUPO and r*Aae*UPO-PaDa-I-H, both of which had previously been shown by us to give the tertiary alcohol *p*-methane 3,8-diol 9 as the sole product.^19^

## Results and discussion

*Dch*UPO was identified from among a number of family I UPOs during a search of the genomic databases using the sequence of *Mro*UPO^18^ as a model. The enzyme has a sequence length of 262 amino acids (Fig. S1), although the predicted native signal sequence accounts for the first seventeen residues. The ‘mature’ protein is predicted to have a molecular weight of approximately 28.7 kDa, confirming it as a member of family I of shorter UPOs. The enzyme displays 29% full length sequence identity with *Mro*UPO (Fig. S2) and also 33% with artUPO and 65% with both *Dca*UPO from *Daldinia caldariorum*^15^ and *Hsp*UPO from *Hypoxylon* sp. EC38,^26^ other family I UPOs that have been the object of attention in recent studies.

### Expression in *Escherichia coli*

As the expression of family I UPOs had been achieved using *E. coli* as a heterologous host,^14–16^ we first ordered a suitably codon-optimised *Dch*UPO gene (Fig. S3) in the standard pET-28a vector, with the native signal peptide removed and equipped with a N-terminal hexa-histidine tag. Expression in *E. coli* was successfully achieved (SI Section S2), and this permitted a three-step purification of *Dch*UPO_bact_ using nickel affinity chromatography, anion exchange and size exclusion (SEC) (Fig. S4) to give homogeneous protein suitable for enzyme assays and crystallisation. The pure protein exhibited a Reinheitszahl value (*R*_*z*_ value, corresponding to the ratio of absorbance at 419 nm and 280 nm) of 3.24, as determined by a UV-Vis scan (Fig. S5). This pure protein was suitable for kinetic assays as had previously been performed for artUPO^16^ and also crystallisation trials.

## Kinetic analysis

We first attempted to establish kinetic parameters for *Dch*UPO_bact_ using the established peroxidase/peroxygenase substrates 2,2′-azino-bis(3-ethylbenzathiazoline-6-sulfonic acid (ABTS, Fig. S6A) and 5-nitro-1,3-benzodioxole (NBD, Fig. S6B and Table 1).^27^*Dch*UPO displayed a comparable *K*_m_ to artUPO for NBD, but a 10-fold higher *k*_cat_. For ABTS kinetic determinations were complicated by pronounced substrate inhibition (Fig. S6A).

**Table 1:** Kinetic parameters for *Dch*UPO compared against those obtained for artUPO_bact_^16^ and *Hsp*UPO^26^ previously

	ABTS		NBD	
	*k*_cat_ (s^−1^)	*K*_m_ (μM)	*k*_cat_ (s^−1^)	*K*_m_ (μM)
*Dch*UPO_bact_	a	a	48	121
artUPO_bact_^16^	45	35	5	106
*Hsp*UPO^26^	17	30	10	18

a Not determined owing to pronounced substrate inhibition (Fig. S6A).

### Structure of *Dch*UPO

**Fig. 2:**
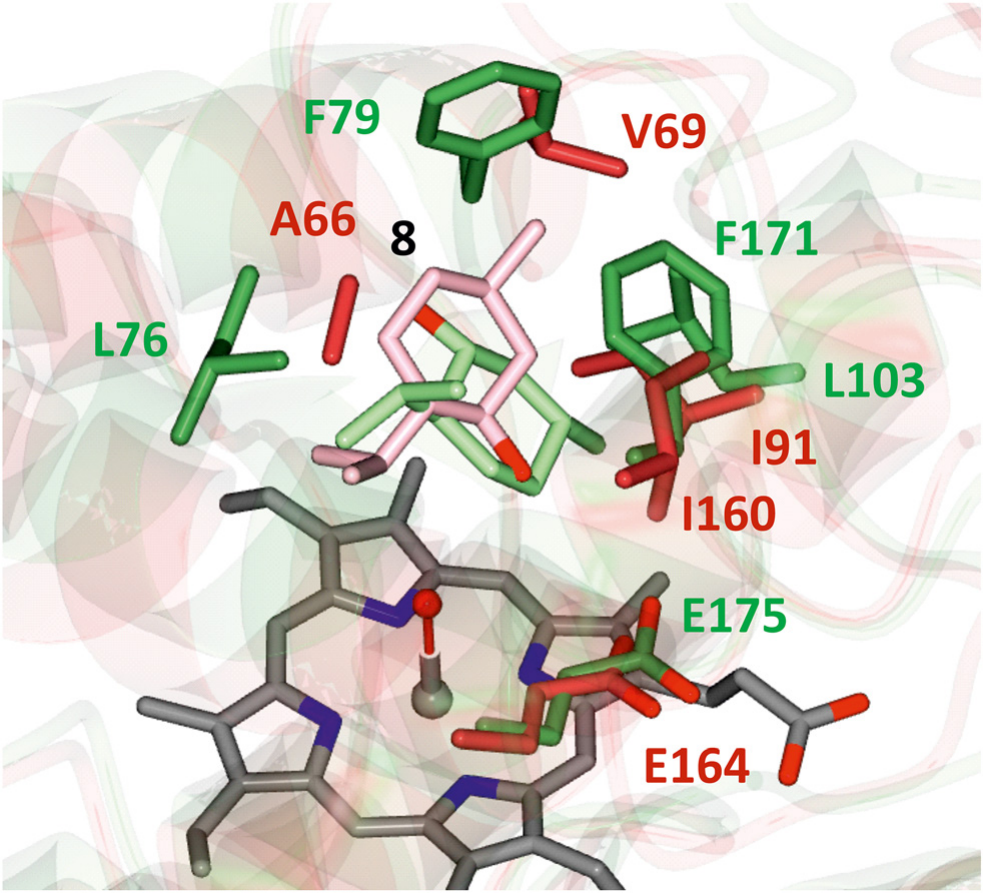
Superimposition of *Dch*UPO and artUPO active sites, each modelled with (–)-menthol 8 using Autodock VINA.^35^ Side chain and (–)-menthol carbon atoms for *Dch*UPO and artUPO models are shown in green and blue respectively.

The structure of *Dch*UPO was determined by X-ray crystallography and refined to a resolution of 1.88 Å (SI Section S4). The crystals were in space group *C*2_1_ and featured two molecules in the asymmetric unit (Fig. 2A). The monomers were complete from residues P21/W22 to P237 with no additional C-terminal density suggesting that residues Q^238^SPGTISKRTEKSSEKRAEKRCPFH^262^ were either too flexible to be modelled or had been cleaved in the crystallisation process. Although there were two molecules in the asymmetric unit, there was no evidence from size exclusion chromatography that *Dch*UPO forms a dimer, and indeed the cysteine residue C232 in the family I artUPO that forms a disulfide bridge with C232 of its neighbour^16^ is absent in the sequence of *Dca*UPO^15^ as in *Hsp*UPO.^26^ The monomer structure (Fig. 2B) was analysed using the DALI server^28^ and found to have the highest homology to *Dca*UPO (PDB code 8IAG; 72% sequence identity using the truncated form of *Dch*UPO; Z-score 38.8; rmsd 0.8 over 222 Cα atoms)^29^ and *Hsp*UPO (7O1X; also 72%; 38.7; 0.5 over 225 Cα atoms).^26^

A comparison of the active site of *Dch*UPO with artUPO, which has been the subject of many studies within our group^16,19–21,30,31^ revealed some similarities but also a number of interesting differences. The cysteine residue C34 and glutamate residue E175 are highly conserved as they are responsible for ligating to the heme iron and for assisting peroxide cleavage respectively. However, several substitutions in the channel approaching the heme in the active site were observed, which alter the topology of this important area for substrate recognition. These include L76 (*Dch*UPO) for A66 (artUPO); F75 (L65), L72 (I62), A178 (F167) G174 (L163), F171 (I160) and V177 (A166) (Fig. 2C). F75 was observed to be in two different conformations in the monomer subunits: ‘closed’ in subunit ‘A’ and ‘open’ in subunit ‘B’, (Fig. 2C), indicative of flexibility and a possible gatekeeper role, governing substrate access to the active site. The differences between *Dch*UPO and artUPO, especially the more sterically restrictive residues F79 and F171, appear to significantly change the topology of the active site tunnel and hence potentially the selectivity of *Dch*UPO when challenged with substrates with a number of oxidatively susceptible C-H bonds.

### Expression in *Komagataella phaffii*

We have previously shown that while the family I UPO artUPO can indeed be expressed in *E. coli*, the recombinant enzyme produced from bacteria is unstable under process conditions when the enzyme is exposed to hydrogen peroxide.^16^ Although the molecular basis for this is ambiguous, it appears that the glycosylation of the UPO confers stability to the enzyme under oxidative stress. We have hence routinely applied artUPO as a crude secretate from *Komagataella phaffii* as a biocatalyst in preparative scale biotransformations.^16,19–21,30,31^ This being the case, we expressed *Dch*UPO also in *K. phaffii*, in an effort to obtain substantial amounts of stable biocatalyst for the purposes of preparative scale biotransformations (SI Section S5). The *Dch*UPO gene was edited to remove the native signal peptide and subcloned into the pPICZαB vector under the control of the AOX promoter, as employed for the artUPO *K. phaffii* expression.^16^ Following transformation and small-scale experiments that employed Western blot analysis to confirm expression, a strain of *P. pastoris* X33 was grown in a fermenter on a 200 mL scale. After 4 d of methanol feeding, the cells were removed and the secretate was concentrated ten-fold using centrifugal concentrators to yield an enzyme preparation suitable for use in biotransformation reactions. The UV-Vis scan for the secretate containing crude *Dch*UPO_yeast_ is shown in Fig. S7 and the preparation presented a Reinheitszahl (*R*_*z*_) value of 0.22.

### *Dch*UPO catalyses a regio-distinct hydroxylation of (–)-menthol

**Scheme 2:**
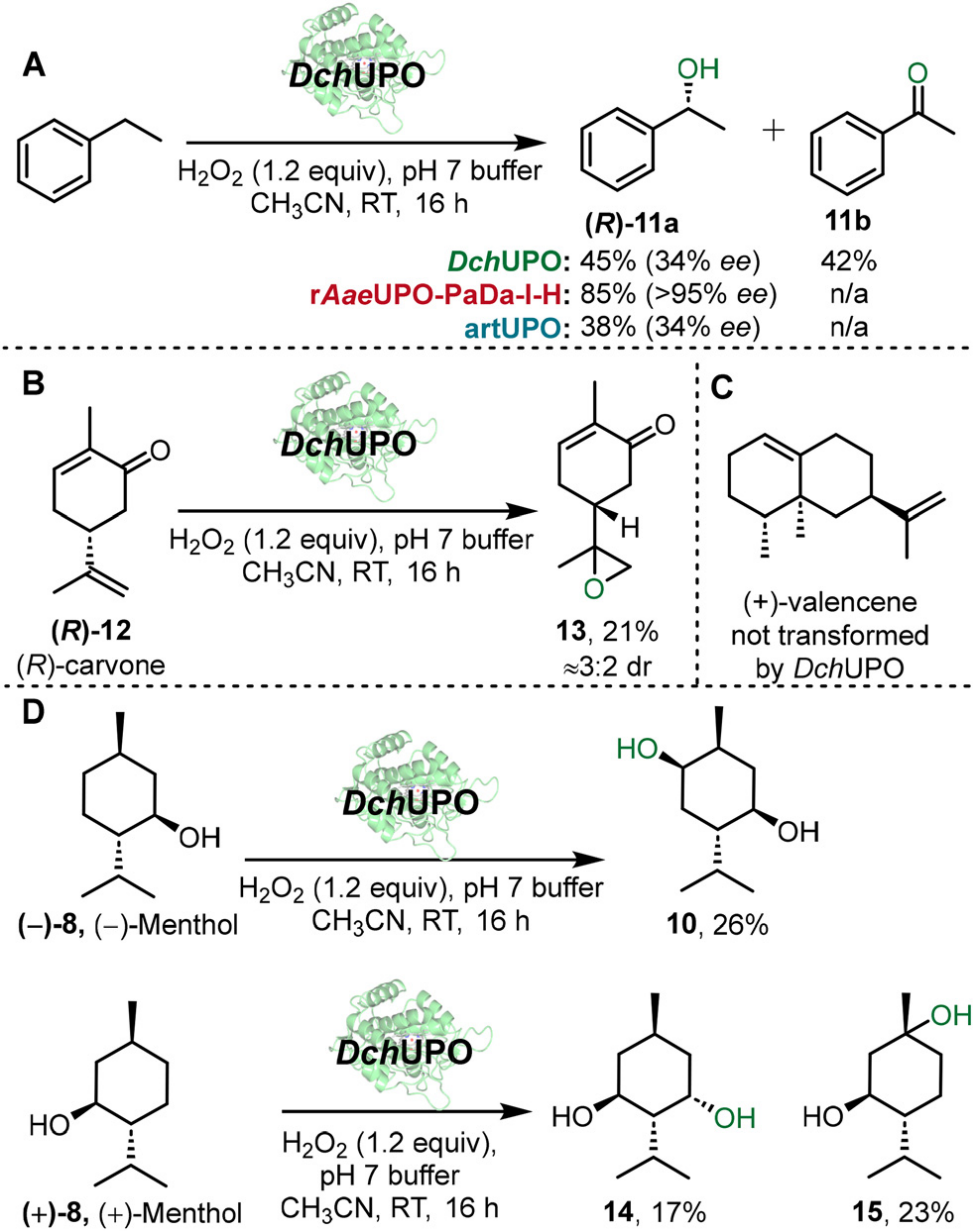
Biotransformations using *Dch*UPO: (A) ethylbenzene; % values refer to conversions measured by GC; (B) (*R*)-carvone; (C) (+)-valencene; (D) (–)- and (+)-menthol. % values in these cases refer to isolated yields. For full preparative details, analytic details and product characterisation data, see SI Section S6.

The different active site topology of *Dch*UPO compared to artUPO suggested that this may give rise to alternate selectivities in biotransformations. We therefore challenged *Dch*UPO with the transformation of ethylbenzene, in addition to selected terpene substrates (SI Section S6). Ethylbenzene is a common substrate used to test C–H oxygenation by UPOs, including in our previous work,^16^ where we describe its conversion into (*R*)-11a using r*Aae*UPO-PaDa-I-H and artUPO in combination with H_2_O_2_. To compare the reactivity of *Dch*UPO to these previously reported biotransformations, ethylbenzene was reacted with *Dch*UPO and 1.2 equivalents of H_2_O_2_ at pH 7. Conversion and ee were measured by GC (Scheme 2A and Fig. S8). Under these conditions, two major products were formed: alcohol (*R*)-11a with 45% conversion and 34% ee, and ketone 11b with 42% conversion. This outcome differs to that seen for UPOs previously tested by us, with the major difference being the formation of significant amounts of the higher oxidation state ketone product 11b. The enantioselectivity for the *Dch*UPO reaction was the same as that observed using artUPO (both 34% ee in favour of the (*R*)-isomer; Fig. S9), which seems reasonable given that both are family I UPOs, although notably, each is far less enantioselective than the family II UPO r*Aae*UPO-PaDa-I-H (>95% ee).^16,32^

Having established that *Dch*UPO is able to promote C–H oxygenation, we moved on to test more complex terpene substrates. In these experiments, a ‘UPO-free’ secretate, derived from fermentation of *K. phaffii* cells that had not been transformed with a UPO gene, was applied in control reactions in parallel, in order to confirm that there was no hydroxylation reaction by *K. phaffii* secretate in the absence of enzyme. In our previous work, we demonstrated that artUPO is capable of transforming both enantiomers of carvone 12 to form epoxide 13 as a mixture of diastereoisomers on a preparative scale (up to 85% isolated yield).^19^ In this study, the preparative scale transformation of (*R*)-carvone (*R*)-12 was tested using *Dch*UPO and 1.2 equivalents of H_2_O_2_, which delivered epoxide 13 in 21% isolated yield, as a ≈3 : 2 mixture of diastereoisomers (Scheme 2B). While the yield of this unoptimised preparative scale biotransformation was low, it is notable that *Dch*UPO was able to promote the same transformation as the established enzyme artUPO. We were also interested to challenge *Dch*UPO with a larger terpene substrate to test its steric limits, and chose (+)-valencene, given that we previously showed that artUPO is capable of converting it into a mixture of three oxidation products.^19^ However, using the same conditions used to transform (*R*)-carvone, no conversion was observed (Scheme 2C). This suggests that *Dch*UPO may be more restrictive than artUPO with respect to its ability to accept larger substrates.^19^

As noted in the Introduction (Scheme 1B), biotransformations of the important flavour and fragrance molecule (±)-menthol 8 using artUPO and r*Aae*UPO-PaDa-I-H had previously been shown by us to give the tertiary alcohol *p*-methane 3,8-diol 9 as the sole product, with the same product being formed from both enantiomers of menthol.^19^ artUPO performs this preparative biotransformation especially well, affording diol 9 in 66% isolated yield in a gram-scale reaction. We therefore challenged *Dch*UPO with both enantiomers of menthol, and interestingly, different products were obtained to those using the previously tested UPOs in both cases (Scheme 2D). For the *Dch*UPO biotransformation of (–)-menthol (–)-8, regio- and stereoselective oxygenation was observed, enabling the isolation of a single product, *cis*-6-hydroxymenthol 10. A completely different regiochemical outcome was observed when the other menthol enantiomer (+)-8 was tested, with diols 14 and 15 isolated in 17% and 23% yields respectively, each as single diastereoisomers. For all three products 10, 14 and 15, the rigidity of the 6-membered ring framework permitted the straightforward assignment of the regio- and stereoselective oxygenation reactions, through consideration of the ^3^*J*_H–H_ coupling constants in their ^1^H NMR data (SI Section S7 for full details).

In its biotransformation of 8, *Dch*UPO was shown to give products of menthol oxygenation which, to our knowledge, had not been previously observed by microbial or enzymatic transformation. The case of the transformation of (−)-8 is especially interesting as only one major product, *cis*-diol 10 was obtained. Miyazawa and co-workers previously reported isolation and biological activity of the *trans*- form of this diol,^33,34^ but we have been unable to find a previous report of the *cis* isomer of (–)-10 in the literature. With (+)-8, other diol products 14 and 15 were obtained. Both of these compounds also appear to be unprecedented in the literature, thus these reactions give access to new products from the commercially important terpenes (–)-and (+)-menthol.

The regio-distinct hydroxylation of (–)-menthol (–)-8 by *Dch*UPO was explored by modelling the substrate into the active site using Autodock VINA^35^ and comparing this with an equivalent model for artUPO, using a structure previously obtained by our group (Fig. 2, SI Section S8).^16^ The lowest energy pose for artUPO with (–)-8 clearly positions the isopropyl group towards the oxygen atom of the Fe=O in the model. However, for *Dch*UPO, this orientation seems to be disfavoured by the presence of F79 and F171, which, in place of V69 and I160 respectively, press the 6-position towards the oxygen of Fe=O. The greater restrictions imposed by the phenylalanine residues in *Dch*UPO are reminiscent of the active site environment in the family II *Aae*UPO, although those Phe residues are not conserved in the same places in *Aae*UPO-PaDa-I.^24^ However, the results together provide further evidence that the presence or absence of large aromatic residues in UPO active sites has a significant influence on reaction selectivity.

## Conclusions

In this paper we have reported the expression and analysis of a family I UPO from *Daldinia childiae* (*Dch*UPO). Family I UPOs are of significant interest as, not only do they perform oxygenation reactions with a range of complementary selectivities to family II UPOs, they also in some cases can be expressed *E. coli*, rather than *Komagataella phaffii*, making them more accessible targets and more amenable to directed evolution. It is useful therefore to identify and characterise new family I UPOs in order to broaden the knowledge of these enzymes but also to provide platforms for evolution of catalysts for transformations with different outcomes to those observed previously for other enzymes.

## Author contributions

G. G., W. P. U., N. P. M. and J. C. designed and supervised experiments. A. M., C. C., K. A. S. C., J. L., J. D., B. M. and M. P. H. R. performed experiments. All authors contributed to the writing of the manuscript.

## Conflicts of interest

There are no conflicts to declare.

## Supplementary Material

CB-OLF-D6CB00141F-s001

## Data Availability

The data supporting this article have been included as part of the supplementary information (SI). Supplemenentary information is available. See DOI: https://doi.org/10.1039/d6cb00141f. Crystallographic data for the *Dch*UPO structure have been deposited in the Protein Data Bank (PDB)] under accession number 9TM7.
