# Supplementary material for: Structure, characterisation and application of an unspecific peroxygenase from Daldinia childiae
Source: RSC Chem Biol. 2026 Jun 11;7(8):1615–20. doi: 10.1039/d6cb00141f (PMC13277406; doi:10.1039/d6cb00141f)
Supplement: Supplementary file 1 [file CB-007-D6CB00141F-s001.pdf]

Supplementary Information (SI) for RSC Chemical Biology.
This journal is © The Royal Society of Chemistry 2026

Structure, Characterisation and Application of an Unspecific Peroxygenase from *Daldinia childiae*

Alexander McKenzie,[a] Claudia Clark,[a] Katy A. S. Cornish,[a,b] Jiacheng Li,[a] Jack Domenech,[a] Benjamin Melling,[a] Miles P. H. Ralston,[a] Jared Cartwright,[b] Nicholas P. Mulholland,[c] William P. Unsworth* [a] and Gideon Grogan*[a]

[a]Department of Chemistry, University of York, Heslington, York YO10 5DD U.K.
[b]Department and Biology, University of York, Heslington, York YO10 5DD U.K.
[c] Syngenta, Jealott's Hill International Research Centre, Bracknell, Berkshire, RG42 6EY (UK).

william.unsworth@york.ac.uk; gideon.grogan@york.ac.uk

Supporting Information

Contents

1. Sequences and Alignments	3–5
2. Expression of *Dch*UPO in *E. coli*	6
3. Kinetics	7
4. Crystallisation and Structure Determination of *Dch*UPO$_{bact}$	8
5. Expression of *Dch*UPO in *Komagataella phaffii*	9–11
6. Biotransformations and product characterisation data	12–17
7. NMR spectra	18–20
8. Modelling using Autodock VINA	21
9. References	21

1. Sequences and Alignments

>*Dch*UPO

MKLVYLSSAVAFGSAIADTAPWEGPGPSDVRGPCPMLNSLANHGFLPHDGKSINVNKTVDAL

SSALNLAPELASFLHSFAVTTNPQPNATTFDLDHLSRHNILEHDGSLSRQDSFFGPADVFNE

AVFNQTKSYWTGDVITIQMAANARVARLMTSNLTNPEYTLSHLGSDFSIGESVAYLSILGSK

ETGEVPKAYVEYLFENERLPYELGFSKMKEPMTESDLEGLMDKLVASQHFPQSPGTISKRTE

KSSEKRAEKRCPFH

Figure S1. Amino acid sequence of *Dch*UPO with signal sequence highlighted in red.

```
DchUPO          -----MKLVYLSSAVAFGSAIADTAPWEGPGPSDVRGPCPMLNSLANHGFLPHDGKSINV
DcaUPO          ------------------------MAPWKAPGPDDVRGPCPMLNTLANHGFLPHDGKNIDV
HspUPO          MKSLSFSLALGFGSTLVYSAPSPSSGWQAPGPNDVRAPCPMLNTLANHGFLPHDGKGITV
artUPO          ---------------SQDIVDFSQHPWKAPGPNDLRSPCPGLNTLANHGFLPRNGRNITI
MroUPO          ------------------AVDFSAHPWKAPGPNDSRGPCPGLNTLANHGFLPRNGRNISV
                                          *:.***.* *.*** **:********::*:.* :
DchUPO          NKTVDALSSALNLAPELASFLHSFAVTTNPQPNATTFDLDHLSRHNILEHDGSLSRQDSF
DcaUPO          NTTVNALSSALNLDDELSRDLHTFAVTTNPQPNATWFSLNHLSRHNVLEHDASLSRQDAY
HspUPO          NKTIDALGSALNIDANLSTLLFGFAATTNPQPNATFFDLDHLSRHNILEHDASLSRQDSY
artUPO          PMIVQAGFDGYNVQPDILILAAKVGLLTSPEP--DTFTLDDLKLHGTIEHDASLSREDFA
MroUPO          PMIVKAGFEGYNVQSDILILAGKIGMLTSREA--DTISLEDLKLHGTIEHDASLSREDVA
                   :.*  .. *:  ::      ..  *. :.    : *:.*. *. :***.****:*
DchUPO          FGPADVFNEAVFNQTKSYWTG-DVITIQMAANARVARLMTSNLTNPEYTLSHLGSDFSIG
DcaUPO          FGPPDVFNAAVFNETKAYWTG-DIINFQMAANALTARLMTSNLTNPEFSMSQLGRGFGLG
HspUPO          FGPADVFNEAVFNQTKSFWTG-DIIDVQMAANARIVRLLTSNLTNPEYSLSDLGSAFSIG
artUPO          LGDNLHFNEAIFNTLANSNPGSDVYNITSAGQVLKDRLADSLARNPNVTNTGKEFTIRTL
MroUPO          IGDNLHFNEAIFTTLANSNPGADVYNISSAAQVQHDRLADSLARNPNVTNTDLTATIRSS
                :*    ** *:*.      .* *:  .  *.:.   **  *   **: : :     :
DchUPO          ESVAYLSILGS--KETGEVPKAYVEYLFENERLPYELGFSKMKEPMTESDLEGLMDKLVA
DcaUPO          ETVCYVTILGS--KETRTVPKAFVEYLFENERLPYELGFKKMKSALTEDELTTMMGEIYS
HspUPO          ESAAYIGILGD--KKSATVPKSWVEYLFENERLPYELGFKRPNDPFTTDDLGDLSTQIIN
artUPO          ESAFYLSVMGN--ATTGEAPKNFVQIFFREERLPIEEGWKRSTTPITSDTLNPIAGQISE
MroUPO          ESAFFLTVMSAGDPLRGEAPKKFVNVFFREERMPIKEGWKRSTTPITIPLLGPIIERITE
                *:. :: ::.        .** :*: :*.:**:* : *:.: . .:*   *  :  .:
DchUPO           SQHFPQ-SPGTISKRTEKSSEKRAEKRCPFH--
DcaUPO           LQHLPESFTKPFAKRSEAPFEKRAEKRCPFH--
HspUPO           AQHFPQ---------SPGKVEKRGDTRCPYGYH
artUPO           ASNWKP-----------NPDQCPWIVLSPNL--
MroUPO           LSDWKP-----------TGDNCGAIVLSPE---
```

Figure S2. Sequence Alignment of Class I UPOs referred to in this study. *Dch*UPO is from *Daldinia childiae*; *Dca*UPO is from *Daldinia caldariorum*; *Hsp*UPO is from *Hypoxolon* sp. EC38; artUPO is 'articial peroxygenase'; *Mro*UPO is from *Marasmius rotula.* The conserved cysteine C34 and glutamate E175 are highlighted in blue and red respectively; the residues lining the active site approach tunnel are highlighted in green. The cysteine that participates in dimer formation on artUPO is highlighted in magenta.

GGATCCGATACAGCTCCTTGGGAAGGGCCAGGACCCTCGGACGTTAGAGGTCCGTGTCCG

ATGCTGAATAGTTTGGCCAACCACGGCTTCCTGCCGCACGACGGCAAGTCCATCAACGTG

AATAAGACTGTAGATGCGTTGTCCTCTGCACTGAACCTCGCCCCTGAGCTGGCGAGCTTC

CTGCATAGCTTCGCTGTGACCACCAATCCGCAACCCAACGCCACCACGTTTGATCTGGAC

CACCTGTCACGTCATAACATTCTGGAGCACGACGGTAGCCTGTCTCGTCAAGATAGCTTC

TTTGGCCCGGCGGATGTGTTTAACGAGGCGGTCTTCAACCAGACCAAAAGCTATTGGACC

GGTGATGTGATCACCATTCAGATGGCGGCAAATGCGCGTGTTGCTCGTCTGATGACCAGC

AATCTGACCAACCCAGAATACACCCTGAGCCACTTGGGCTCCGACTTCAGCATTGGTGAG

AGCGTTGCGTACTTATCTATCTTGGGTTCCAAAGAGACGGGCGAGGTGCCGAAAGCATAT

GTTGAATACCTGTTTGAGAACGAACGTCTTCCGTATGAACTCGGCTTCTCCAAGATGAAA

GAGCCGATGACGGAAAGCGACTTGGAGGGTCTGATGGATAAACTGGTTGCTTCCCAGCAT

TTTCCGCAAAGCCCGGGTACTATCAGCAAGCGCACCGAAAAGTCTAGCGAAAAGCGCGCA

GAAAAACGCTGCCCGTTTCATTAAAAGCTT

Figure S3. Gene encoding *Dch*UPO used in the preparation of the pET-28a construct for expression in *E. coli*.

2. Expression and Purification of *Dch*UPO from *E. coli*

The gene encoding *Dch*UPO in a pET-28a vector was used to transform OverExpress™ C41(DE3) Chemically Competent Cells (Sigma-Aldrich) according to the manufacturer's instructions and plated onto low-salt LB Agar with kanamycin at a concentration of 30 µg mL^{-1} and incubated at 37 °C overnight. Transformants were inoculated into 60 mL LB broth containing 30 µg mL^{-1} kanamycin and incubated at 37 °C overnight with shaking at 180 rpm. 10 mL of each of these starter cultures were used to inoculate 500 mL each of sterile ZYM-5052 autoinduction medium, supplemented with kanamycin (100 µg mL^{-1}), $FeSO_4$ (1.2 mM), and 5-aminolevulinic acid (5-ALA, 1 mM). Each flask was inoculated with 10 mL of the overnight cultures and incubated at 16 °C with shaking at 180 rpm for 96 h. Cells were then pelleted by centrifugation at 5,400 ×*g* for 20 min at 4 °C and resuspended on ice in ~200 mL of a resuspension buffer containing 20 mM phosphate, 300 mM NaCl, 10% (v/v) glycerol and 30 mM imidazole at pH 7.8 (Buffer 'A'). Cell lysis was then carried out using a CF2 cell disruptor (Constant Systems Ltd.) at 27kPa and 4 °C. Cell lysate was clarified by centrifugation at 20,000 ×*g* for 40 min at 4 °C. The resulting supernatant was collected and subjected to chromatographic protein purification.

Purification of ***Dch*UPO$_{bact}$**

Soluble lysate fractions were filtered using 0.45 µm syringe filters, and loaded *via* peristaltic pump onto a HisTrap FF Crude 5 mL column (Cytiva) equilibrated with Buffer 'A'. columns were washed with 10 column volumes (CVs) of Buffer 'A' and eluted over a 10 CV gradient using a limit Buffer 'B' which was formulated as for Buffer 'A' but contained 500 mM imidazole. The cleanest fractions, as established by SDS-PAGE analysis (**Figure S4A**) were pooled and dialysed against Buffer 'C' (Buffer 'A' without NaCl or imidazole) to remove salt. They were then loaded onto HiTrap QHP 5 mL column (Cytiva), which was then washed with 10 CV Buffer 'C', and eluted over 20 CV using a gradient with limit buffer 'D' (Buffer 'A' with 500 mM NaCl and no imidazole). The cleanest fractions, as established by SDS-PAGE analysis (**Figure S4B**) were pooled and concentrated to a volume of approximately 2 mL, after which they were subjected to size exclusion chromatography (SEC) using a HiLoad Superdex 75 pg 16 × 600 mm column (Cytiva) equilibrated into SEC Buffer 'E' (Buffer 'A' without imidazole). Fractions containing *Dch*UPO, as determined by SDS-PAGE analysis (**Figure S4C**) were then retained for crystallisation and kinetics studies.

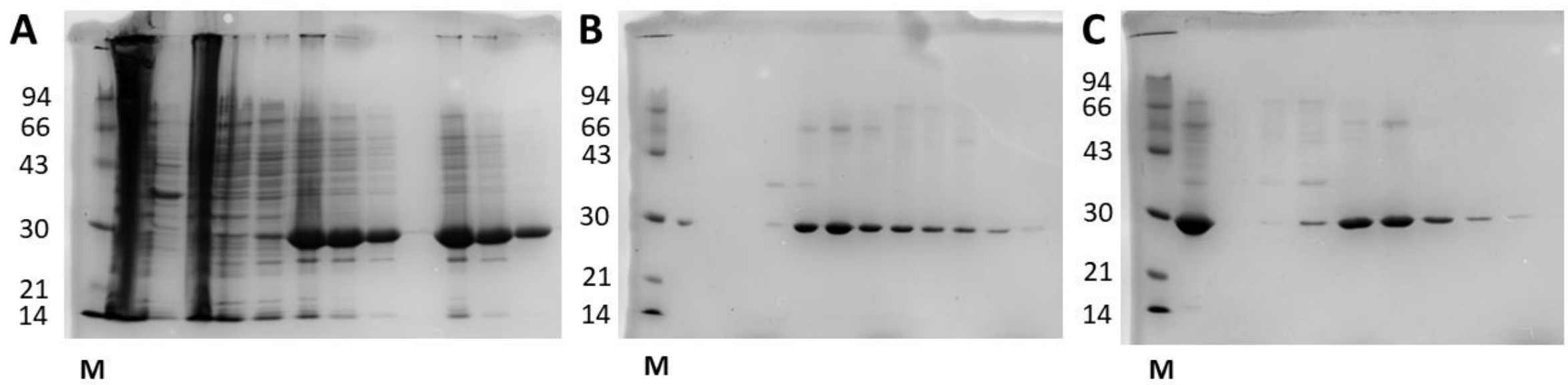

Figure S4. SDS-PAGE analysis of three-step purification of *Dch*UPO$_{bact}$. **A**: NiNTA chromatography; **B**: Anion exchange chromatography; **C**: Size exclusion chromatography. **M** = low molecular weight markers with MW in kDa shown on the y-axes.

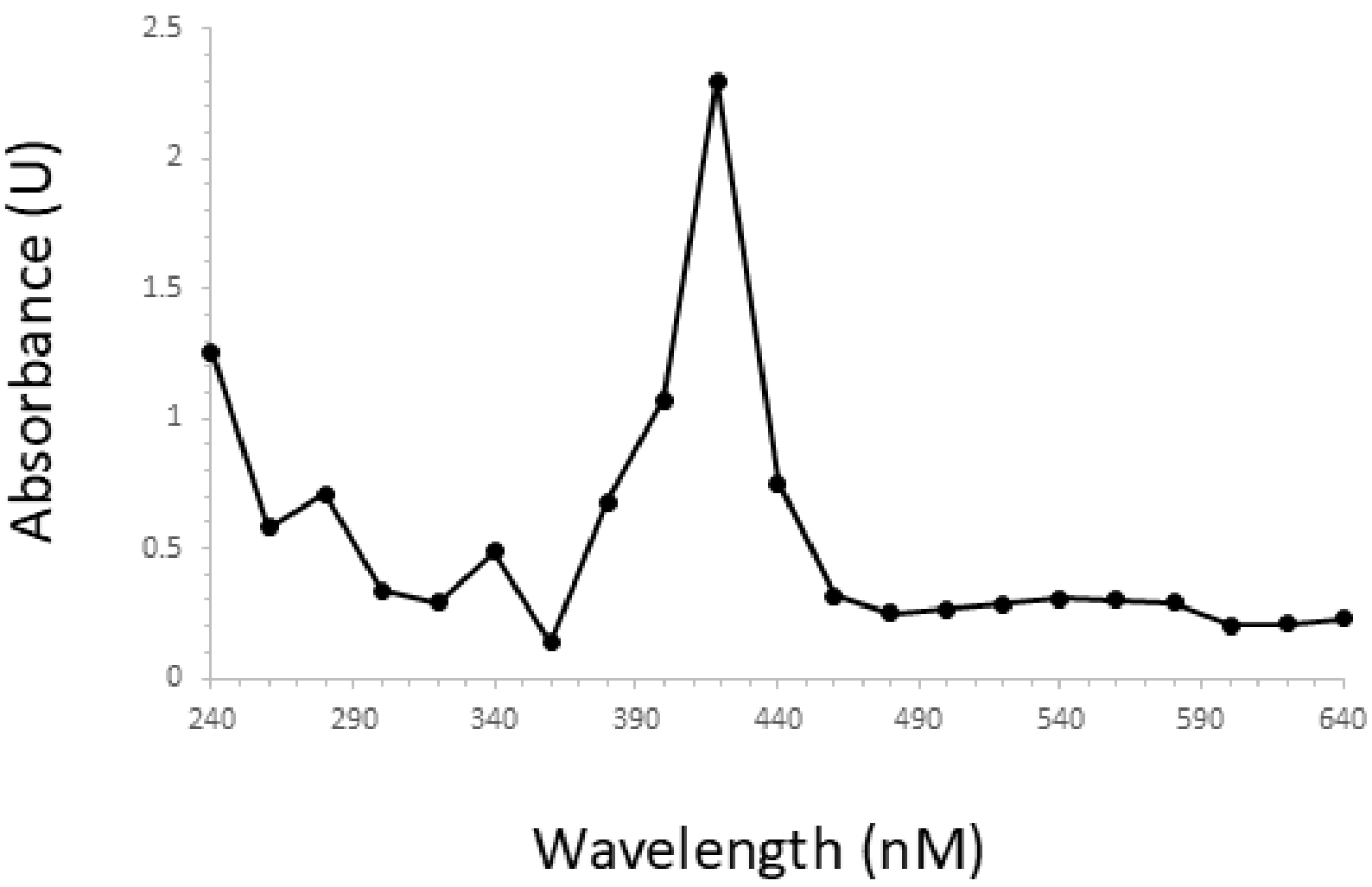

Figure S5. UV-Vis scan of purified *Dch*UPO$_{\mathbf{bact}}$ showing a Reinheitszahl value (R_z value, corresponding to the ratio of absorbance at 419 nm and 280 nm) of 3.24.

3. Kinetics

Kinetic constants for peroxidase and peroxygenase activity of *Dch*UPO$_{bact}$ were determined in UV-Vis assays using the substrates 2,2'-azino-bis(3-ethylbenzthiazoline-6-sulfonic acid) (ABTS) and 1,2-(methylenedioxy-4-nitrobenzene) (NBD) respectively. The reactions for both substrates were carried out on a 1 mL scale in 1 mL quartz cuvettes. For the ABTS assays, the reactions contained 75 mM citrate buffer at pH 4.0, ABTS at concentrations of 0, 10, 20, 50, 100, 250, 500, and 1000 M and 17.2 µg of purified *Dch*UPO$_{bact}$. Reactions were initiated by the addition of H_2O_2 to a final concentration of 2 mM. For the NBD assays, the reactions contained 50 mM potassium phosphate buffer at pH 7.0,

NBD with final concentrations of 0, 100, 250, 500, 750 and 1000 μM in acetonitrile (5% of the final volume), 17.2 μg of purified *Dch*UPO$_{bact}$ Reactions were again initiated by the addition of H_2O_2 to a final concentration of 2 mM. ABTS and NBD reactions were monitored for 1 min by absorbance at 418 nm for ABTS and 420 nm for NBD. Monitoring the rate of reaction at different concentrations generated Michaelis-Menten curves for both ABTS and NBD for *Dch*UPO$_{bact}$ (**Figures S6A** and **S6B** respectively).

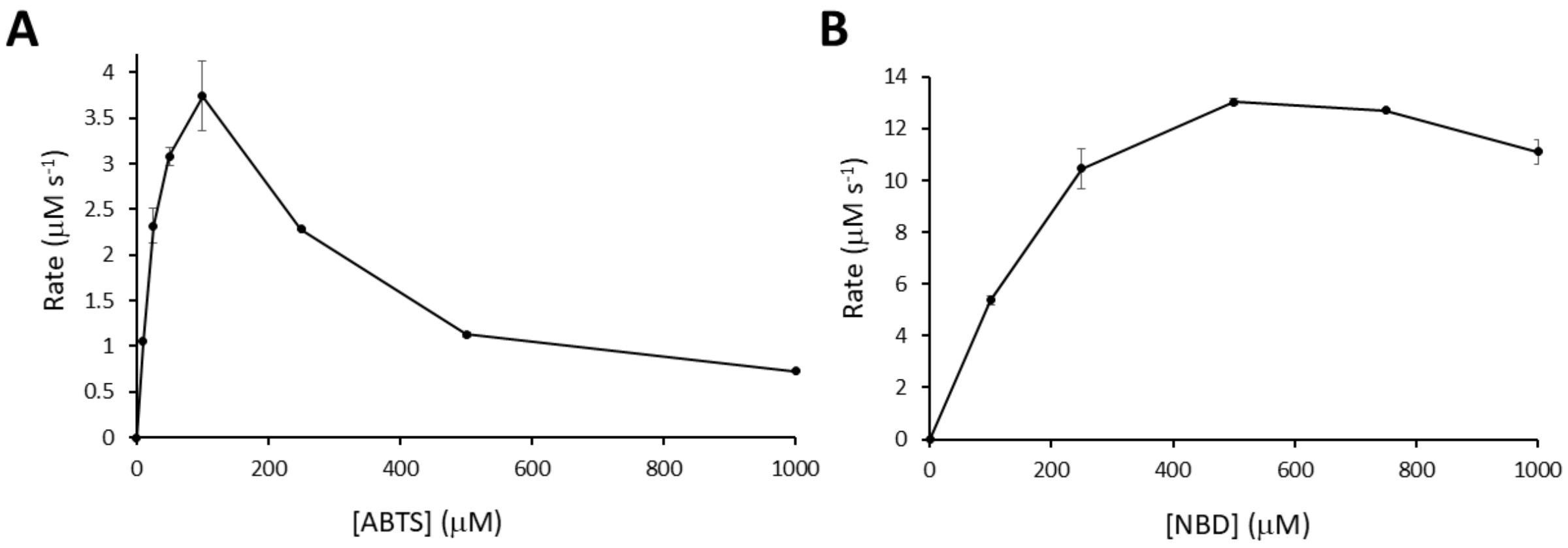

Figure S6. Kinetic plots for *Dch*UPO assayed against **A**: ABTS and **B:** NBD. Each point represents the average of technical duplicate measurements.

4. Crystallisation and Structure Determination of *Dch*UPO$_{bact}$

Purified *Dch*UPO$_{bact}$ was concentrated to 12 mg mL^{-1} and subjected to crystallisation using a Mosquito robot and a range of commercially available crystallisation screens in a 96-well plate format. The best crystals were obtained from conditions containing 0.2 M calcium chloride dihydrate, 0.1 M MES buffer at pH 6.0 and 20 % (w/v) PEG 6000.

Data collection, structure solution and refinement

Crystals of *Dch*UPO$_{bact}$ were flash-cooled using liquid nitrogen without extra cryo-protectant. The datasets described in this report were collected at Diamond Light Source, Didcot, Oxfordshire, U.K. on Beamline I03. Data were processed and integrated using XDS[1] and scaled using SCALA[2] included in the Xia2 processing system.[3] Data collection statistics are provided in **Table S1**. Crystals of *Dch*UPO$_{bact}$ were obtained in space group $C2_1$, with two molecules in the asymmetric unit. The structure was solved by molecular replacement using MOLREP[4] with one monomer of *Dch*UPO generated using Alphafold[5] as the model. The structure was built and refined using iterative cycles in Coot[6] and REFMAC,[7] employing local non-crystallographic symmetry (NCS) restraints in the refinement cycles. The final *Dch*UPO$_{bact}$ structure exhibited % R_{cryst}/R_{free} values of 21.2 and 25.2. Refinement statistics for the structures are

presented in **Table S1**. Structure factors and coordinate files for *Dch*UPO$_{bact}$ have been deposited in the Protein DataBank (PDB) with the accession code **9TM7**.

Table S1. Data Collection and Refinement Statistics for *Dch*UPO$_{bact}$. Numbers in brackets refer to data for highest resolution shells.

	***Dch*UPO$_{bact}$**
Beamline	Diamond I03
Wavelength (Å)	0.976250
Resolution (Å)	40.64-1.88 (1.92-1.88)
Space Group	$C\ 2_1$
Unit cell (Å)	A = 81.60; b = 78.05; c = 62.59<br>[illegible] 90.00°; [illegible] 95.18°
No. of molecules in the asymmetric unit	1
Unique reflections	31736 (2030)
Completeness (%)	99.7 (99.9)
R_{merge} (%)	0.14 (1.18)
$R_{p.i.m.}$	0.08 (0.72)
Multiplicity	7.0 (7.2)
$<I/\sigma(I)>$	7.4 (1.5)
Overall *B* factor from Wilson plot (Å^2)	21
$CC_{1/2}$	1.00 (0.69)
R_{cryst}/R_{free} (%)	21.2/25.2
r.m.s.d 1-2 bonds (Å)	0.008
r.m.s.d 1-3 angles (°)	1.67
Avge main chain B (Å^2)	36
Avge side chain B (Å^2)	38
Avge water B (Å^2)	38

5. Expression of *Dch*UPO in *Komagataella phaffii*

Cloning & generation of recombinant strains

The gene encoding *Dch*UPO was codon optimised for expression in *Komagataella phaffii* and synthesised by Twist Bioscience. The gene was then amplified from its carrier plasmid by PCR and inserted by In Fusion cloning into the pPICZα B vector (Invitrogen), adapted to include an N-terminal His_6-tag downstream of the alpha mating factor (MFα) secretion signal sequence. The gene was designed not to include the native *Dch*UPO signal peptide, in order for the expressed UPOs to be secreted under the influence of the MFα secretion signal. The gene sequence is shown in **Table S2**.

Table S2: *Dch*UPO gene sequence for expression in *P. pastoris*.

*Dch*UPO	GATACAGCTCCTTGGGAAGGGCCAGGACCCTCGGACGTTAGAGGTCCGTGTCCGATGCTG<br>AATAGTTTGGCCAACCACGGCTTCCTGCCGCACGACGGCAAGTCCATCAACGTGAATAAG<br>ACTGTAGATGCGTTGTCCTCTGCACTGAACCTCGCCCCTGAGCTGGCGAGCTTCCTGCAT<br>AGCTTCGCTGTGACCACCAATCCGCAACCCAACGCCACCACGTTTGATCTGGACCACCTG<br>TCACGTCATAACATTCTGGAGCACGACGGTAGCCTGTCTCGTCAAGATAGCTTCTTTGGC<br>CCGGCGGATGTGTTTAACGAGGCGGTCTTCAACCAGACCAAAAGCTATTGGACCGGTGAT<br>GTGATCACCATTCAGATGGCGGCAAATGCGCGTGTTGCTCGTCTGATGACCAGCAATCTG<br>ACCAACCCAGAATACACCCTGAGCCACTTGGGCTCCGACTTCAGCATTGGTGAGAGCGTT<br>GCGTACTTATCTATCTTGGGTTCCAAAGAGACGGGCGAGGTGCCGAAAGCATATGTTGAA<br>TACCTGTTTGAGAACGAACGTCTTCCGTATGAACTCGGCTTCTCCAAGATGAAAGAGCCG<br>ATGACGGAAAGCGACTTGGAGGGTCTGATGGATAAACTGGTTGCTTCCCAGCATTTTCCG<br>CAGAGCCCGGGTACTATCAGCAAGCGCACCGAAAAGTCTAGCGAAAAGCGCGCAGAAAAA<br>CGCTGCCCGTTTCAT

The recombinant *Dch*UPO plasmid was used to transform Stellar™ Competent *E. coli* cells (Takara Bio) and plated overnight at 37 °C on low-salt LB agar plates containing 25 µg mL^{-1} zeocin. Transformant colonies were used to inoculate 10 mL starter cultures of low-salt LB with 25 µg mL^{-1} zeocin and grown overnight at 37 °C with shaking at 180 rpm. Recombinant plasmid DNA was extracted and purified

using the QIAGEN plasmid *Plus* Midi Kit, and the *Dch*UPO sequence was confirmed by Sanger sequencing (Source BioScience).

Purified plasmid DNA was linearised with *PmeI* restriction enzyme and used to transform *P. pastoris* strain X-33 (Invitrogen) *via* electroporation. The transformed cells were plated on a Yeast extract Peptone Dextrose plate with 100 µg mL^{-1} zeocin (YPDZ) and incubated at 30 °C for 3 d. Colonies were streaked onto fresh YPDZ plates and incubated for a further 3 d at 30 °C. Clean single colonies were then used to inoculate 5 mL Buffered Glycerol-complex Medium (BMGY) containing 1% yeast extract; 2% peptone; 100 mM potassium phosphate pH 6.0; 1.34% (w/v) yeast nitrogen base; 4×10^{-5} % biotin and 1% glycerol, and grown for 24 h at 30 °C with shaking at 220 rpm. Glycerol stocks for the transformed *Dch*UPO *P. pastoris* strains were prepared using a 1:1 mixture of BMGY culture with 50 % (v/v) sterile glycerol and stored at −70 ºC.

Fermentation & *Dch*UPO production

A 0.5 L MiniBio fermenter (Applikon) was charged with 200 mL of a basal salts medium containing 26.7 mL L^{-1} H_3PO_4, 85 % (w/v); 1.17 g L^{-1} $CaSO_4\cdot2H_2O$; 18.2 g L^{-1} K_2SO_4; 14.9 g L^{-1} $MgSO_4\cdot7H_2O$; 4.13 g L^{-1} KOH; 40.0 g L^{-1} glycerol, and 4.35 mL L^{-1} of PTM_1 trace salts containing 6.0 g L^{-1} $CuSO_4\cdot5H_2O$; 0.08 g L^{-1} NaI; 3.0 g L^{-1} $MnSO_4\cdot H_2O$; 0.2 g L^{-1} $Na_2MoO_4\cdot2H_2O$; 0.02 g L^{-1} H_3BO_3; 0.5 g L^{-1} $CoCl_2$; 20.0 g L^{-1} $ZnCl_2$; 65 g L^{-1} $FeSO_4\cdot7H_2O$; 0.2 g L^{-1} biotin; 5.0 mL L^{-1} H_2SO_4). The following parameters were set for the fermentation: temperature at 30 °C, condenser at 70 %, pH at 5.0, base pump output at 25 %, airflow at 200 mL min^{-1}, dissolved oxygen (DO) at 35 % relative to air saturation and maintained by stirrer limits set to 500–1250 rpm plus O_2 supplementation. The pH was adjusted to 5.0 using 28% ammonium hydroxide solution.

A glycerol stock of the *Dch*UPO *P. pastoris* strain was streaked onto a Yeast extract Peptone Dextrose (YPD) plate with 100 µg mL^{-1} zeocin and incubated at 30 °C for 3 d. Two colonies were selected and grown in 2×10 mL Buffered Glycerol-complex Medium (BMGY) containing 1 % yeast extract; 2 % peptone; 100 mM potassium phosphate pH 6.0; 1.34% (w/v) yeast nitrogen base; 4×10^{-5} % biotin and 1 % glycerol for 24 h at 30 °C with shaking at 220 rpm. The starter cultures were pooled and added to the conditioned fermentation vessel, and the culture grown until all the glycerol had been consumed (determined by DO spike; approximately 20 h). The cell biomass was further increased by a fed-batch phase on 50 % (w/v) glycerol containing 12 mL L^{-1} of filter sterilised PTM_1 trace salts, at a feed rate of 18 mL h^{-1} L^{-1} of initial fermentation volume for 4 h. To induce expression of UPO, a 100% methanol feed with PTM_1 salts (12 mL L^{-1}) was then added at 3.6 mL h^{-1} L^{-1} of initial fermentation volume for 3 h, before increasing to 4.2 mL h^{-1} L^{-1} for 24 h. When the culture had adapted to the methanol feed

rate, had a steady DO % and a fast DO spike after stopping methanol addition, the feed rate was increased to 7.2 mL h^{-1} L^{-1} of initial fermentation volume for 24 h, and further increased to 6.8 mL h^{-1} L^{-1} for the remainder of the cultivation. The cultures were harvested at 96 h from the start of methanol addition and centrifuged at 5,000 × *g* for 20 min to remove the cells, with the UPO located in the secretate. The secretate was further clarified at 10,000 × *g* for 20 min and concentrated approximately 8-fold by spin concentration at 4000 × *g* (Pierce Protein Concentrator PES, 10K MWCO). The concentrated liquid *Dch*UPO secretate was stored at −70 ºC and used crude in all reactions described.

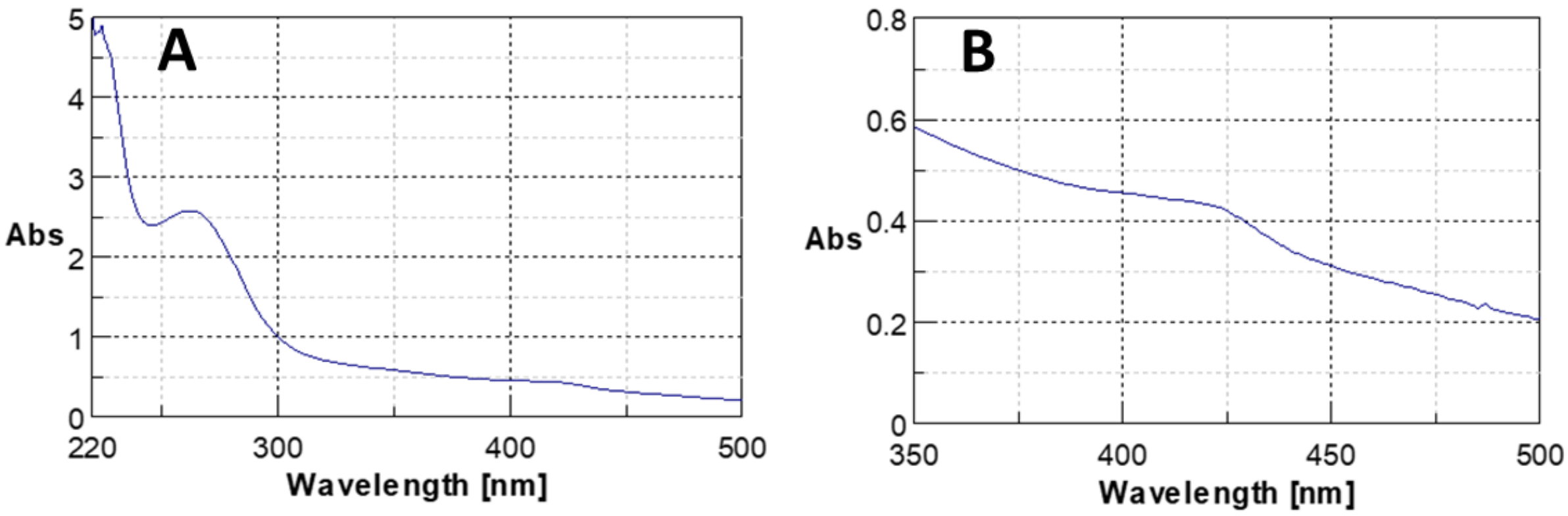

Figure S7. **A**: UV-Vis absorbance scan of secretate of *Komagataella phaffii* containing crude *Dch*UPO; **B**: Detail of same. The spectra show a Reinheitszahl value (R_z value, corresponding to the ratio of absorbance at 419 nm and 280 nm) of 0.22. Secretate was diluted 1 in 20 with PBS for UV-Vis scans, and background corrected against PBS.

6. Biotransformations and product characterisation data

*Dch*UPO biotransformation of ethylbenzene

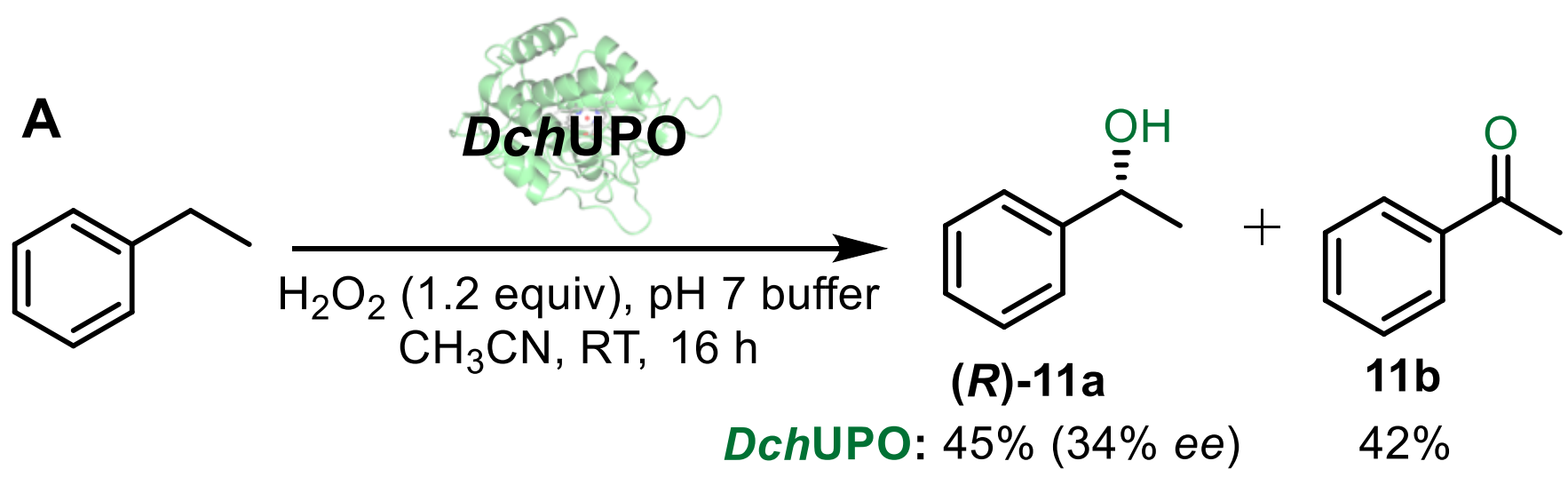

To a round bottomed flask containing a magnetic stirring bar was added *Dch*UPO (3.0 mL), H_2O (15 mL) and KPI buffer (30 mL, 100 mM, pH 7.0). To the solution, ethylbenzene (74 µL , 0.60 mmol, 1.0 equiv., final concentration 10 mM) in MeCN (6 mL) was then added. Next, 6 mL of a 120 mM H_2O_2 solution (prepared from 73 µL of 30% H_2O_2 in 6 mL deionised water, 0.72 mmol, 1.2 equiv.) was added

over a 10 h period, using a syringe pump. After the H_2O_2 addition was complete, the reaction was then stirred at room temperature for a further 6 h. The reaction mixture was then extracted with diethyl ether (3 × 40 mL). The combined organic phases were then washed with brine (40 mL), dried over anhydrous $MgSO_4$ and carefully concentrated *in vacuo* to give the crude product mixture, which was then submitted to GC analysis to obtain conversion and *ee*.

GC: Conversions: **45%** 1-Phenylethanol, **42%** acetophenone (HP-5 column, 120 °C isothermal) *ee*: **34%** assigned as **(*R*)-** based on literature for *Aae*UPO (BGB-175 column, 70 °C for 1 min then ramped to 120 °C over 10 min then isothermal).

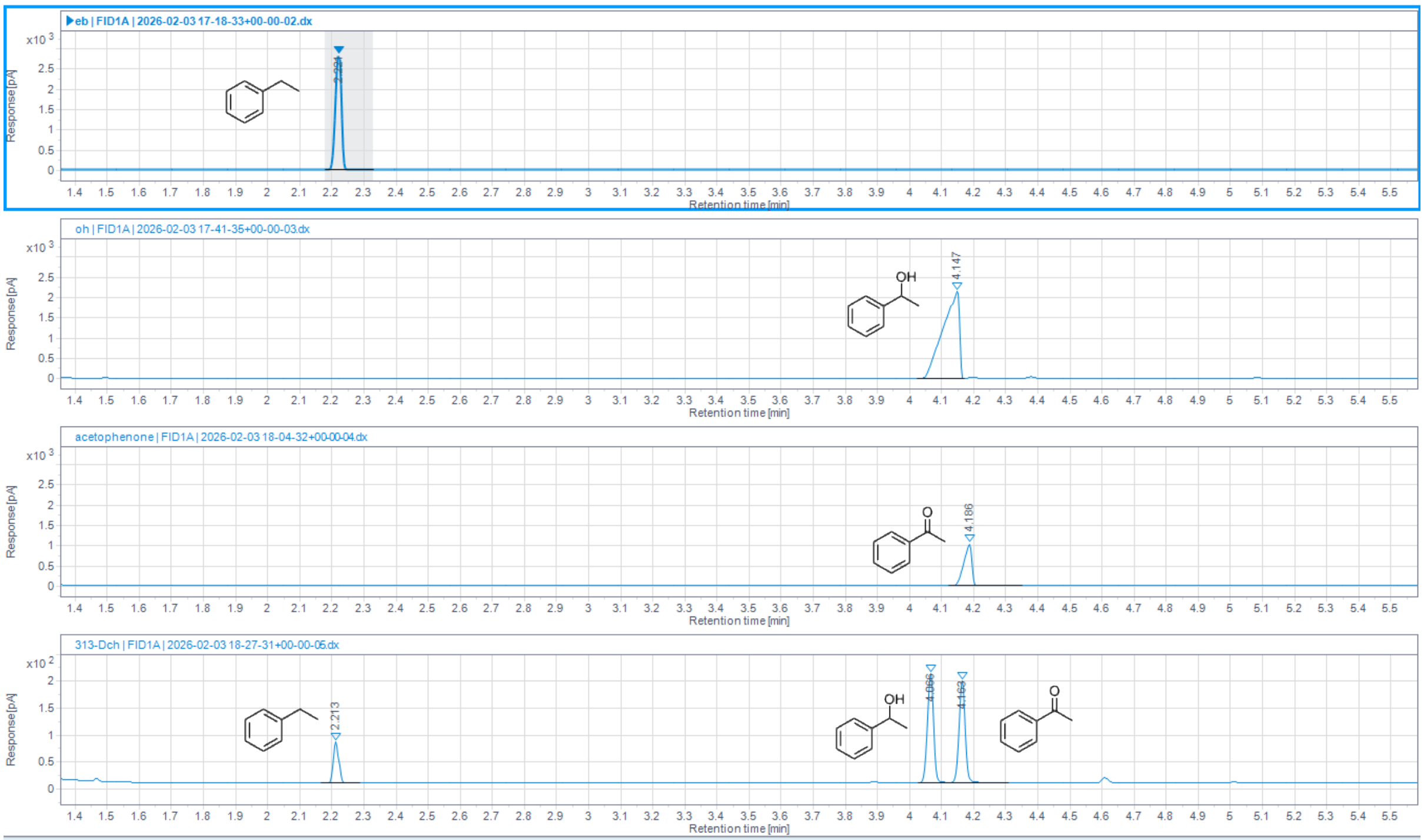

#	Name	RT (min)	Area%	Area	Height	Height%	Amount	Concentration	Start time (min)	End time (min)
1		2.213	13.602	84.758	76.025	16.19			2.167	2.287
2		4.066	44.596	277.898	203.299	43.29			4.027	4.124
3		4.163	41.803	260.494	190.244	40.51			4.124	4.307

Figure S8. GC traces for ethyl benzene (top), alcohol **11a** (2nd), ketone **11b** (3rd) and the biotransformation of ethyl benzene with *Dch*UPO and peak areas (4th and 5th). HP-5 column, 120 °C isothermal.

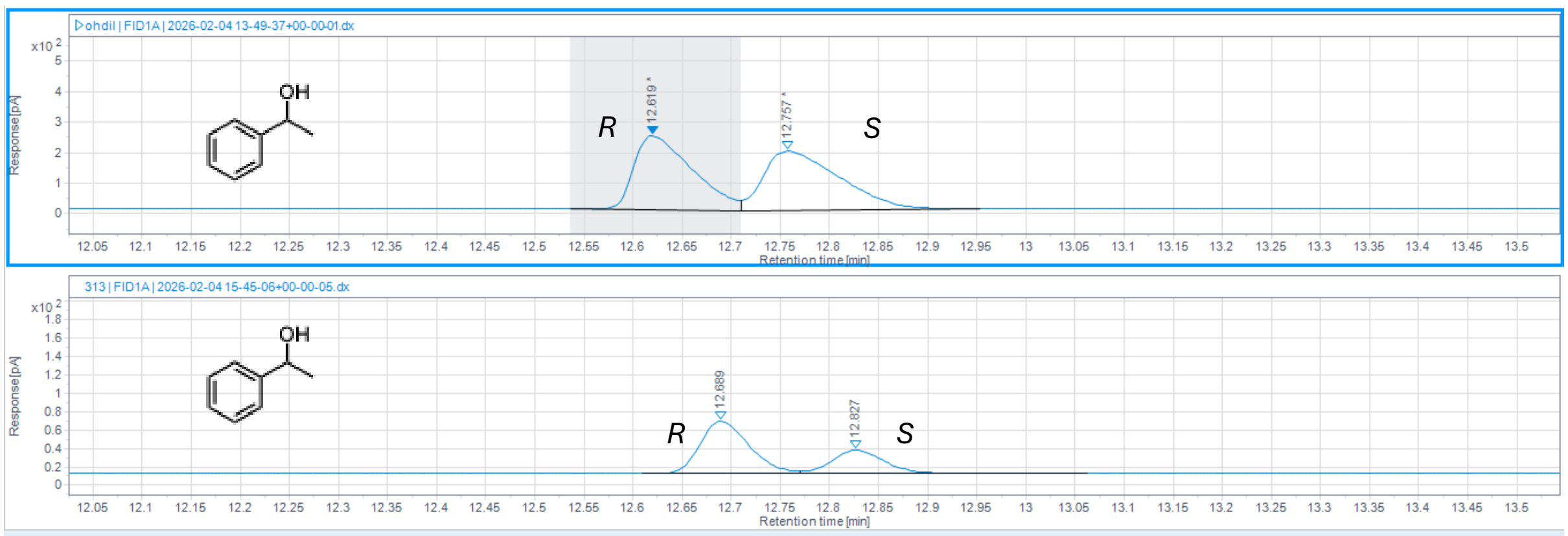

#	Name	RT (min)	Area%	Area	Height	Height%	Amount	Concentration	Start time (min)	End time (min)
1		12.619	49.125	1002.784	242.196	55.60			12.537	12.711
2		12.757	50.875	1038.526	193.431	44.40			12.711	12.953

#	Name	RT (min)	Area%	Area	Height	Height%	Amount	Concentration	Start time (min)	End time (min)
1		12.689	67.231	198.331	57.178	69.15			12.609	12.771
2		12.827	32.769	96.667	25.513	30.85			12.771	13.063

Figure S9: Chiral GC traces and peaks areas for racemic (top) and enantiomerically enriched **11a** (2nd). BGB-175 column, 70 °C for 1 min then raised to 120 °C over 10 min then isothermal.

***Dch*UPO biotransformation of (*R*)-carvone ((*R*)-12)**

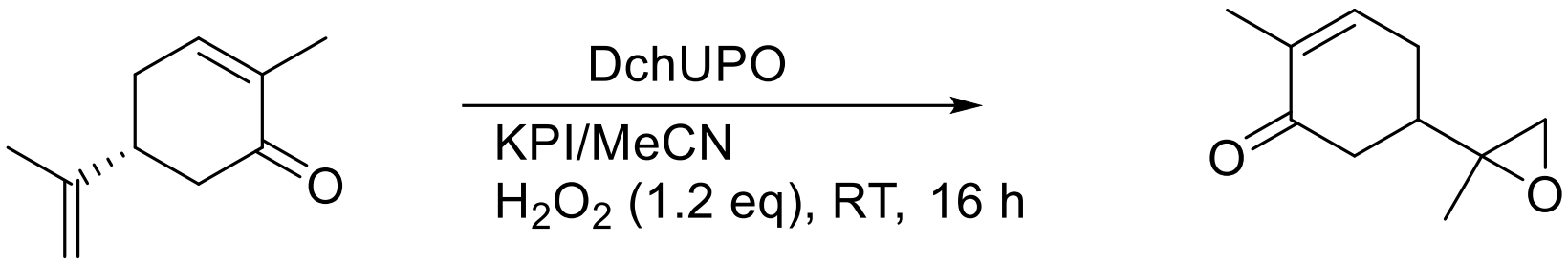

(*R*)-Carvone

13, 21% yield
1:0.64 dr

To a round bottomed flask containing a magnetic stirring bar was added *Dch*UPO (1.0 mL), H_2O (3 mL) and KPI buffer (10 mL, 100 mM, pH 7). To the solution, the *R*-carvone (30.0 mg, 0.2 mmol, 1.0 equiv., final concentration 10 mM) in MeCN (4 mL) was added. Next, 2 mL of a 120 mM H_2O_2 solution (prepared from 24 µL 30% H_2O_2 in 2 mL deionised water, 0.24 mmol) was added over a 10 h period, using a syringe pump. After the H_2O_2 addition was complete, the reaction was then stirred at room temperature for a further 6 h. The reaction mixture was then extracted with ethyl acetate (3 × 20 mL). The combined organic phases were then washed with brine (20 mL), dried over anhydrous $MgSO_4$ and concentrated *in vacuo* to give the crude product mixture, which was purified by flash column chromatography on silica gel (eluent: hexane: ethyl acetate 4:1 – 2:1) to provide the 2-methyl-5-(2-methyloxiran-2-yl)cyclohex-2-en-1-one (7.0 mg, 21% yield) as colourless oil, as a mixture of diastereomers (A:B 1:0.64 ratio) which was determined by ^{1}H NMR. ^{1}H NMR (400 MHz, CHLOROFORM-*D*) δ 6.73 (s, 1H, A/B), 2.70 (d, *J* = 4.5, 1H, A),2.67 (d, *J* = 4.5, 1H, B), 2.60 (d, *J* = 4.5, 1H, B), 2.57 (d, *J* =

4.5, 1H, A), 2.55 – 2.47 (m, 1H, A/B), 2.45 – 2.35 (m, 1H, A/B), 2.30 – 2.15 (m, 3H, A/B), 1.78 – 1.76 (m, 3H, A/B), 1.32 (s, 3H, A), 1.30 (s, 3H, B). Spectroscopic data matched those reported for the analogous artUPO transformation.[8]

(1*R*,2*S*,4*R*,5*S*)-2-isopropyl-5-methylcyclohexane-1,4-diol (10)

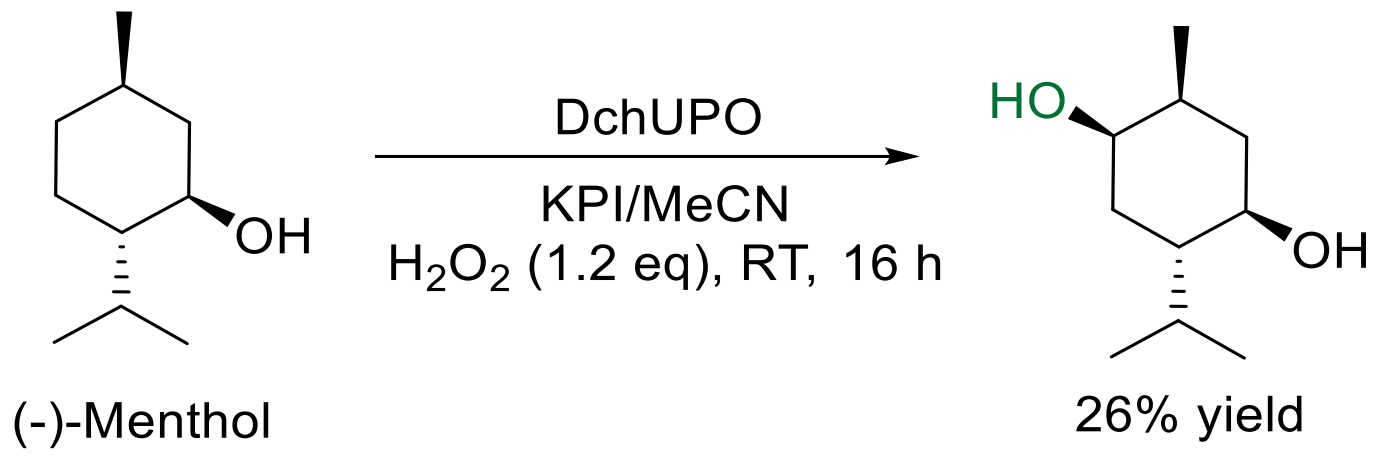

To a round bottomed flask containing a magnetic stirring bar was added *Dch*UPO (1.0 mL), H_2O (3 mL) and KPI buffer (10 mL, 100 mM, pH 7). To the solution, (–)-menthol (32 mg, 0.2 mmol, 1.0 equiv., final concentration 10 mM) in MeCN (4 mL) was added. Next, 2 mL of a 120 mM H_2O_2 solution (prepared from 24 µL 30% H_2O_2 in 2 mL deionised water, 0.24 mmol) was added over a 10 h period, using a syringe pump. After the H_2O_2 addition was complete, the reaction was then stirred at room temperature for a further 6 h. The reaction mixture was then extracted with ethyl acetate (3 × 20 mL). The combined organic phases were then washed with brine (20 mL), dried over anhydrous $MgSO_4$ and concentrated *in vacuo* to give the crude product mixture, which was purified by flash column chromatography on silica gel (eluent: hexane: ethyl acetate 4:1) to provide the title compound **10** (9.0 mg, 26% yield) as white solid.

Data for 10

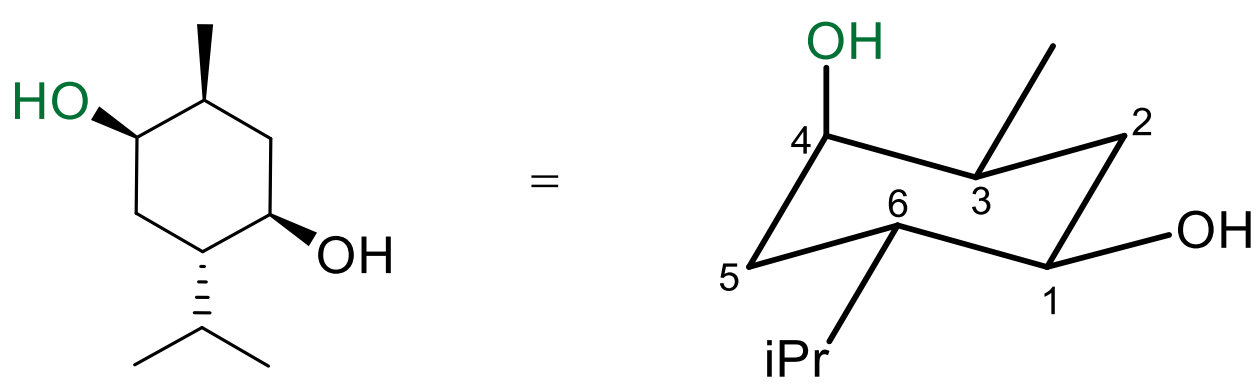

Data for **10:** White solid; (9.0 mg, 26% yield); m.p. 148−149 °C; ^{1}H NMR (400 MHz, CHLOROFORM-*D*) δ 3.81 (m, 1H, H4), 3.43 (app td, *J* = 10.5, 4.5 Hz, 1H, H1), 2.21 (app pd, *J* = 7.1, 3.2 Hz, 1H, H6), 1.81 – 1.67 (m, 2H, $H2_{eq}$ and $H5_{eq}$), 1.65 – 1.52 (m, 2H, H3 and iPr-CH), 1.47 – 1.36 (m, 1H, $H2_{ax}$), 1.27 (d, *J* = 12.1 Hz, 2H, OH), 1.19 (dd, *J* = 13.5, 3.2 Hz, 1H, $H5_{ax}$), 0.99 (d, *J* = 7.1 Hz, 3H, Me), 0.93 (d, *J* = 7.1 Hz, 3H, iPr-CH_3), 0.82 (d, *J* = 7.1 Hz, 3H, iPr-CH_3); ^{13}C NMR (101 MHz, CHLOROFORM-*D*) δ 71.4 (C4), 70.0 (C1), 43.0 (C6), 37.9 (C5), 35.2 (C3), 30.8 (C2), 25.5 (iPr), 20.9 (iPr), 17.9 (Me), 16.1 (iPr); HRMS (ESI) calcd. for $C_{10}H_{19}O_2$ (M-):171.1390, found 171.1380; IR (film): v_{max}/cm^{-1} 3299, 2944, 2867, 1450, 1025, 998.

(1*R*,2*R*,3*R*,5*R*)-2-isopropyl-5-methylcyclohexane-1,3-diol (14) and (1*R*,3*S*,4*R*)-4-isopropyl-1-methylcyclohexane-1,3-diol (15)

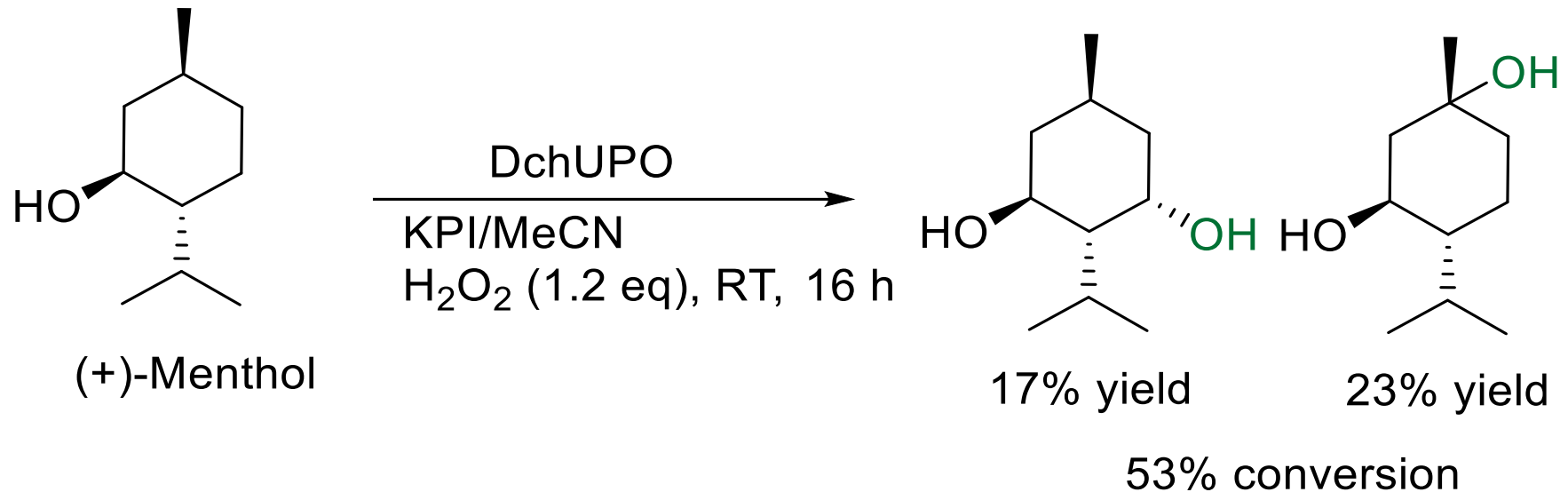

To a round bottomed flask containing a magnetic stirring bar was added *Dch*UPO (1.0 mL), H_2O (3 mL) and KPI buffer (10 mL, 100 mM, pH 7). To the solution, (+)-menthol (32 mg, 0.2 mmol, 1.0 equiv., final concentration 10 mM) in MeCN (4 mL) was added. Next, 2 mL of a 120 mM H_2O_2 solution (prepared from 24 µL 30% H_2O_2 in 2 mL deionised water, 0.24 mmol) was added over a 10 h period, using a syringe pump. After the H_2O_2 addition was complete, the reaction was then stirred at room temperature for a further 6 h. The reaction mixture was then extracted with ethyl acetate (3 × 20 mL). The combined organic phases were then washed with brine (20 mL), dried over anhydrous $MgSO_4$ and concentrated *in vacuo* to give the crude product mixture, which was purified by flash column chromatography on silica gel (eluent: hexane: ethyl acetate 4:1) to afford **14** (6.0 mg, 17% yield) as a colourless solid and **15** (8.0 mg, 23% yield) as colourless crystals.

Data for 14

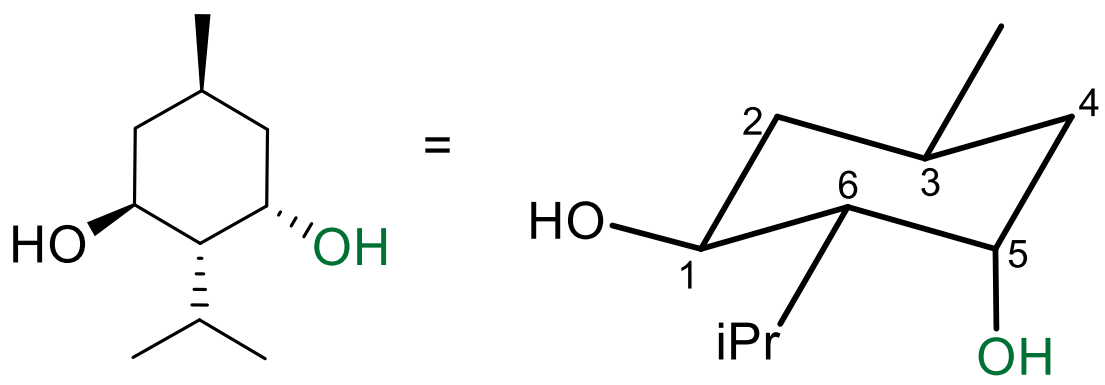

Data for **14**: Colourless solid; m.p. 88−90 °C; ^{1}H NMR (400 MHz, CHLOROFORM-*D*) δ 4.27 – 4.20 (m, 1H, H5), 3.96 (app td, *J* = 10.6, 4.7 Hz, 1H, H1), 2.15 (app pd, *J* = 7.0, 4.7 Hz, 1H, H6), 2.06 – 1.99 (m, 1H, $H2_{eq}$), 1.99 – 1.89 (m, 1H, H3), 1.73 (dtd, *J* = 13.9, 3.6, 2.1 Hz, 1H, $H4_{eq}$), 1.28 – 1.21 (m, 2H, OH), 1.15 – 1.12 (m, 1H, iPr), 1.10 (m, 4H, iPr and $H4_{ax}$), 1.08 (d, *J* = 4.7 Hz, 3H, iPr), 1.03 – 0.95 (m, 1H, $H2_{ax}$), 0.93 (d, *J* = 6.6 Hz, 3H, Me); ^{13}C NMR (101 MHz, CHLOROFORM-*D*) δ 69.6 (C5), 68.2 (C1), 52.9 (C6), 45.3 (C2), 42.9 (C4), 27.4 (iPr), 25.3 (C3), 22.0 (Me), 21.1 (iPr), 20.3 (iPr); HRMS (ESI) calcd. for $C_{10}H_{19}O_2$ (M-): 171.1390, found 171.1399; IR (film): v_{max}/cm^{-1} 3394, 3325, 2923, 2952, 1455, 1027.

Data for 15

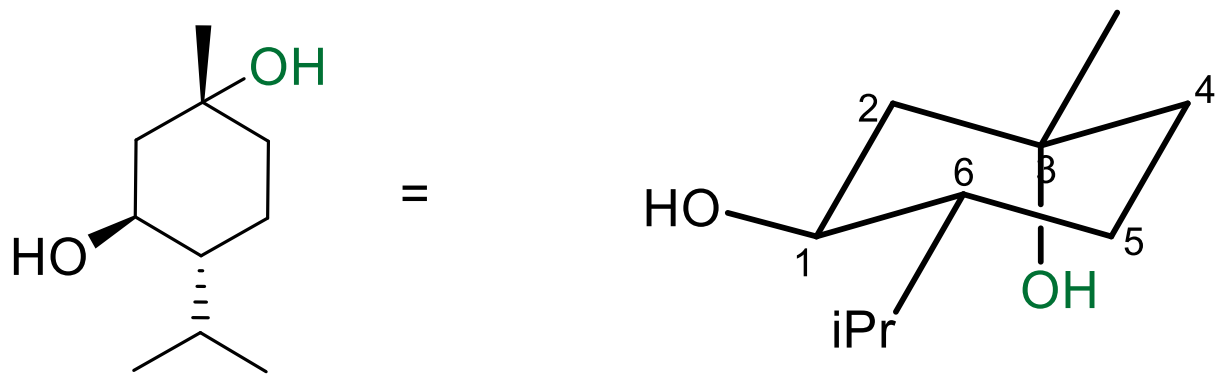

Data for **15**: Colourless crystal; (8.0 mg, 23% yield); m.p. 135−136 °C; ^{1}H NMR (400 MHz, CHLOROFORM-*D*) δ 3.76 (br t, *J* = 10.5, 1H, H1), 2.18 (app pd, *J* = 7.0, 2.6 Hz, 1H, H6), 2.00 (ddd, *J* = 10.5, 4.5, 2.6 Hz, 1H, H2$_{eq}$), 1.63 (dt, *J* = 10.5, 2.6 Hz, 1H, H4$_{eq}$), 1.51 – 1.45 (m, 1H, H5$_{eq}$), 1.40 – 1.35 (m, 2H, iPr and OH), 1.34 – 1.32 (m, 1H, H4$_{ax}$), 1.25 (s, 4H, Me and H2$_{ax}$), 1.16 – 1.09 (m, 1H,H5$_{ax}$), 1.06 (s, 1H, OH), 0.95 (d, *J* = 7.0 Hz, 3H, iPr), 0.85 (d, *J* = 7.0 Hz, 3H, iPr); ^{13}C NMR (101 MHz, CHLOROFORM-*D*) δ 71.6 (C3), 68.6 (C1), 50.1 (C6), 48.3 (C2), 38.5 (C4), 31.7 (Me), 25.9 (iPr), 21.1 (iPr), 19.0 (C5), 16.3 (iPr); HRMS (ESI) calcd. for $C_{10}H_{19}O_2$ (M-): 171.1390, found 171.1396; IR (film): v_{max}/cm^{-1} 3292, 2954, 2934, 1154, 1051.

7. NMR spectra

(1*R*,2*S*,4*R*,5*S*)-2-isopropyl-5-methylcyclohexane-1,4-diol (10)

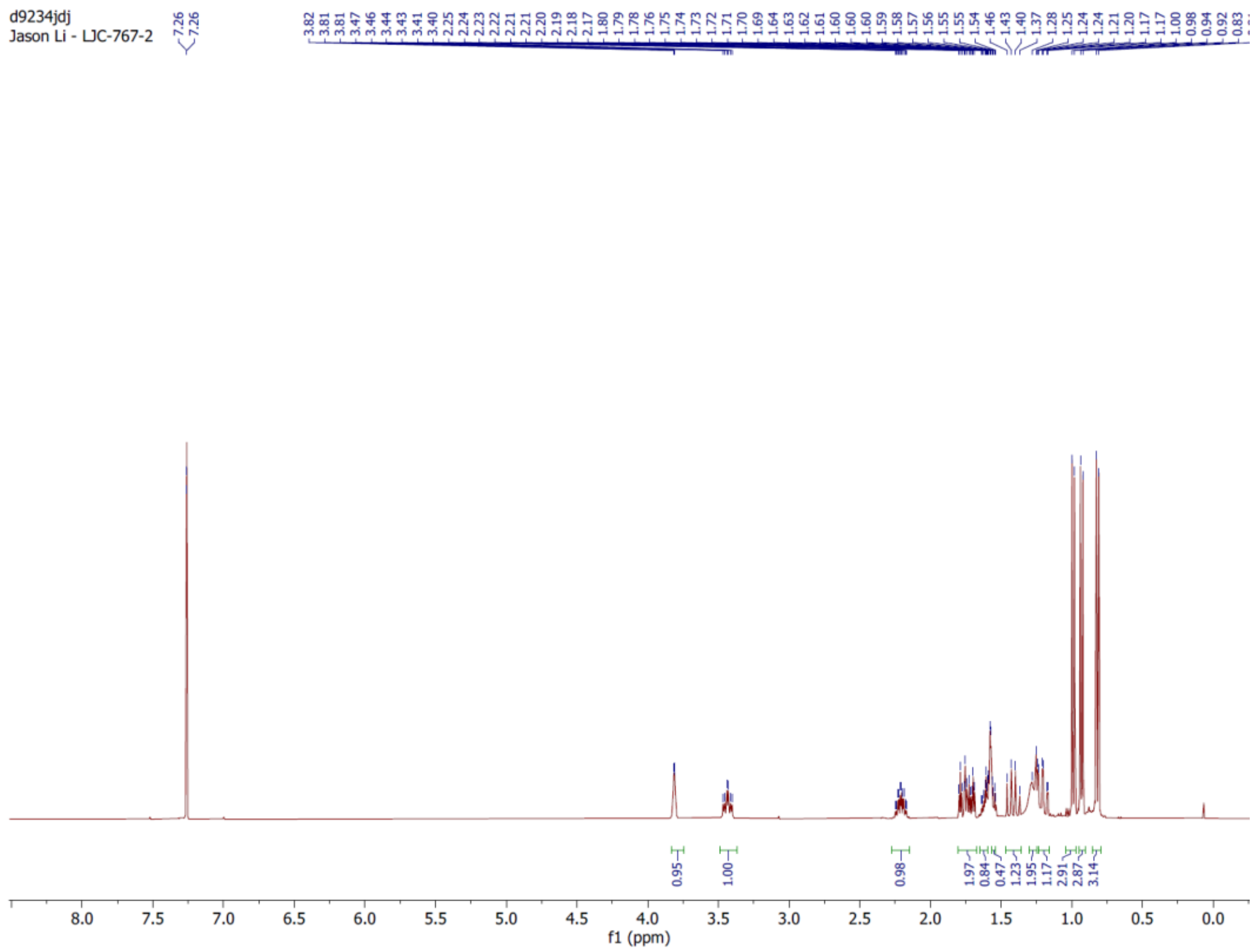

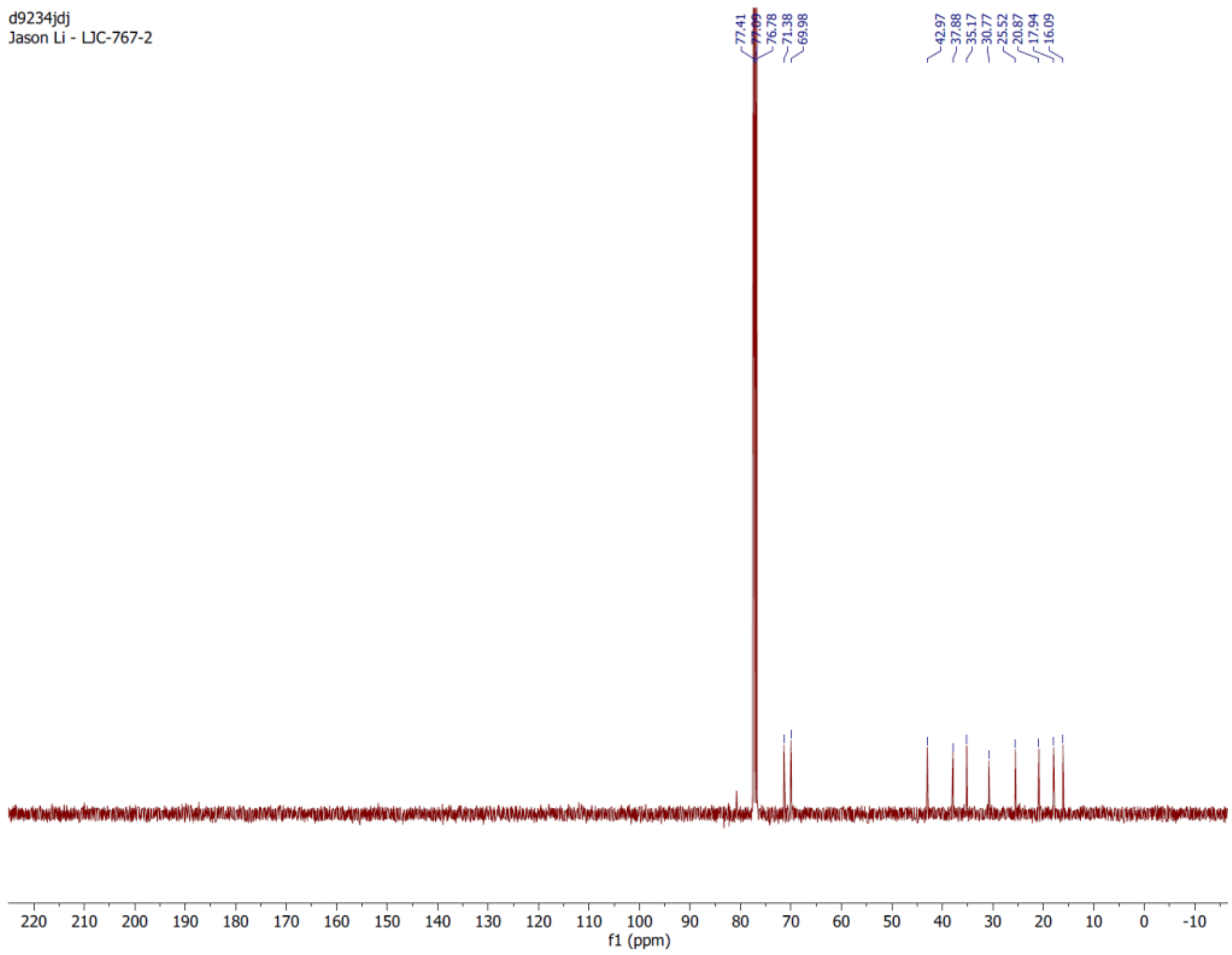

(1*R*,2*R*,3*R*,5*R*)-2-isopropyl-5-methylcyclohexane-1,3-diol (14)

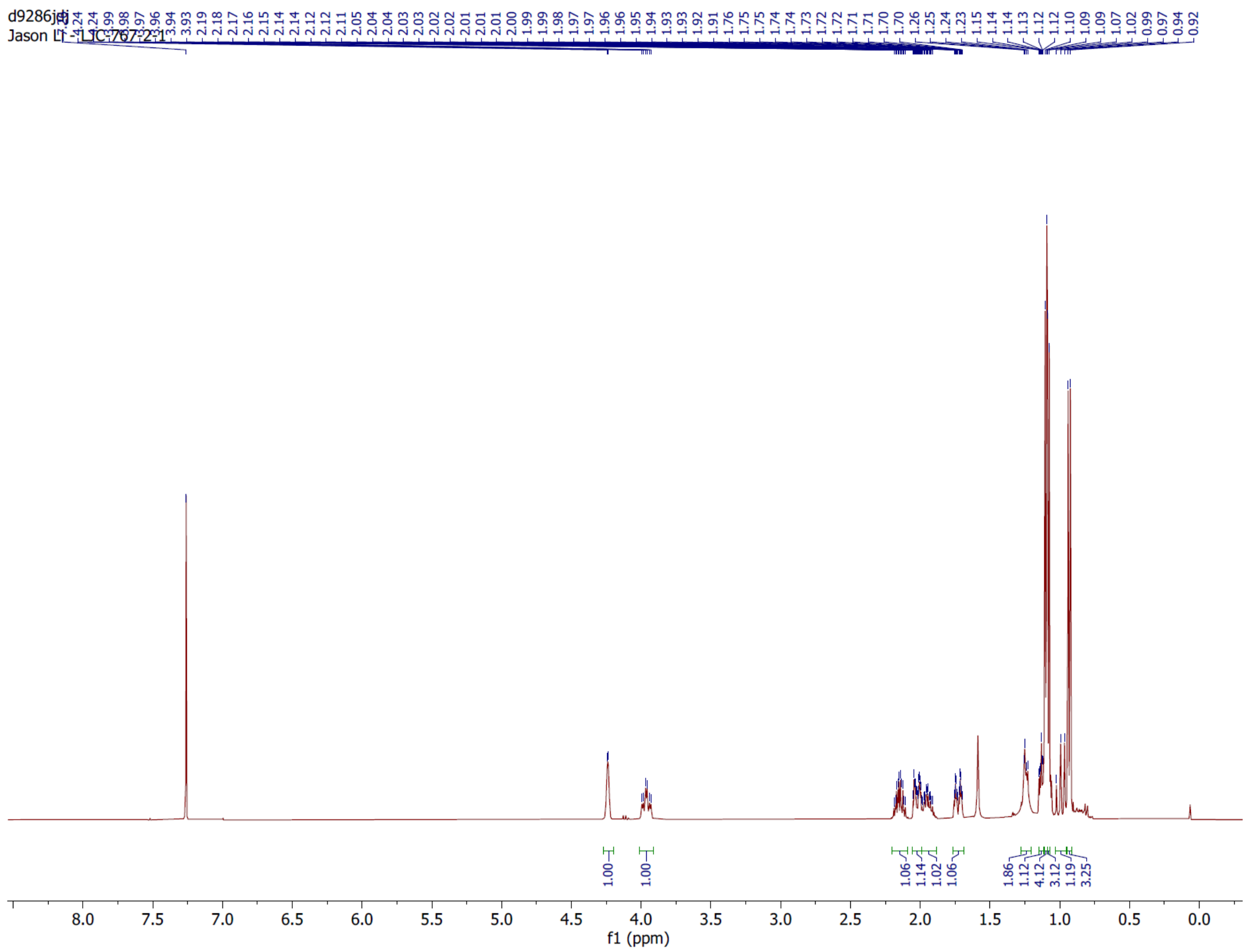

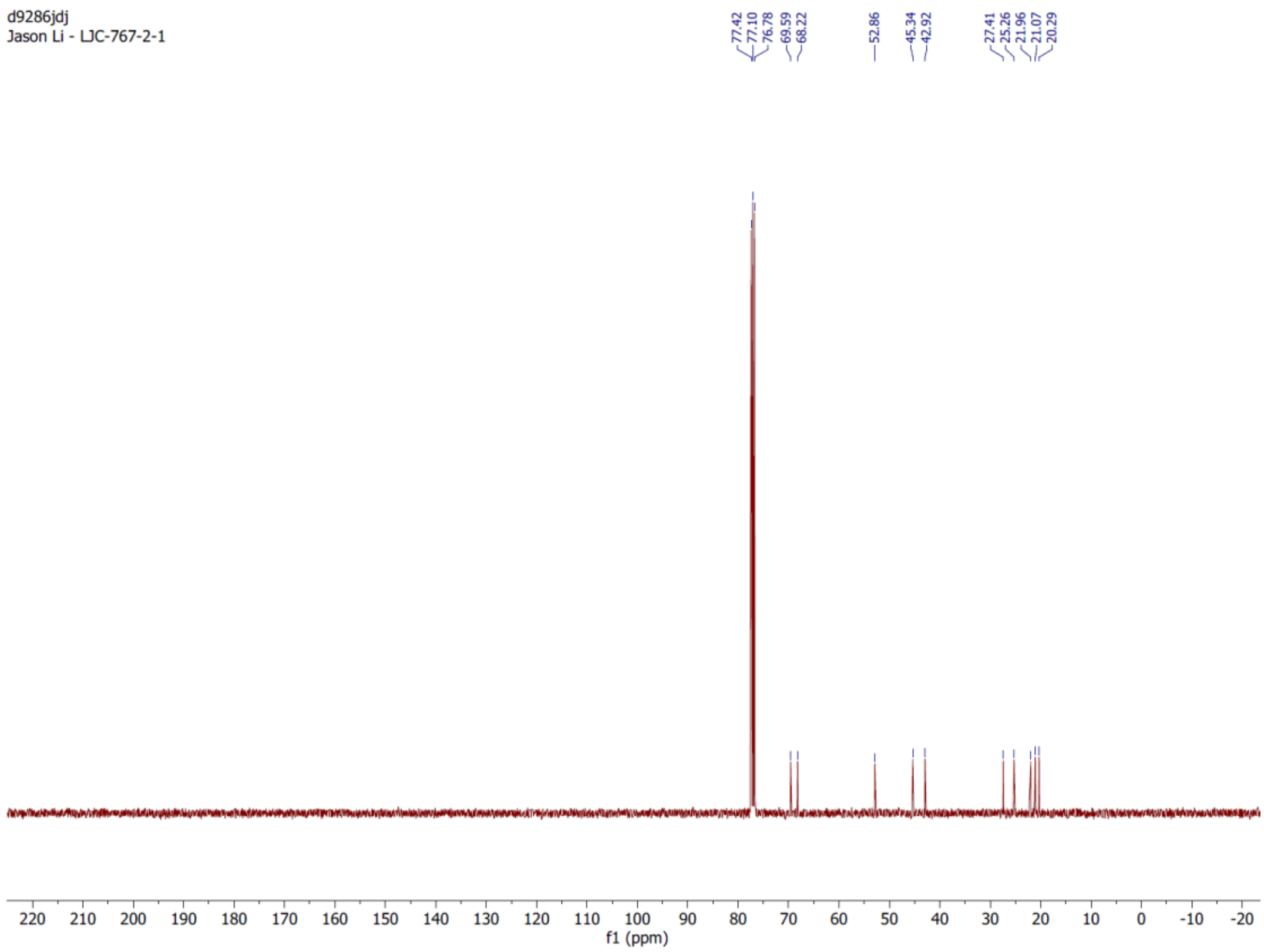

(1*R*,3*S*,4*R*)-4-isopropyl-1-methylcyclohexane-1,3-diol (15)

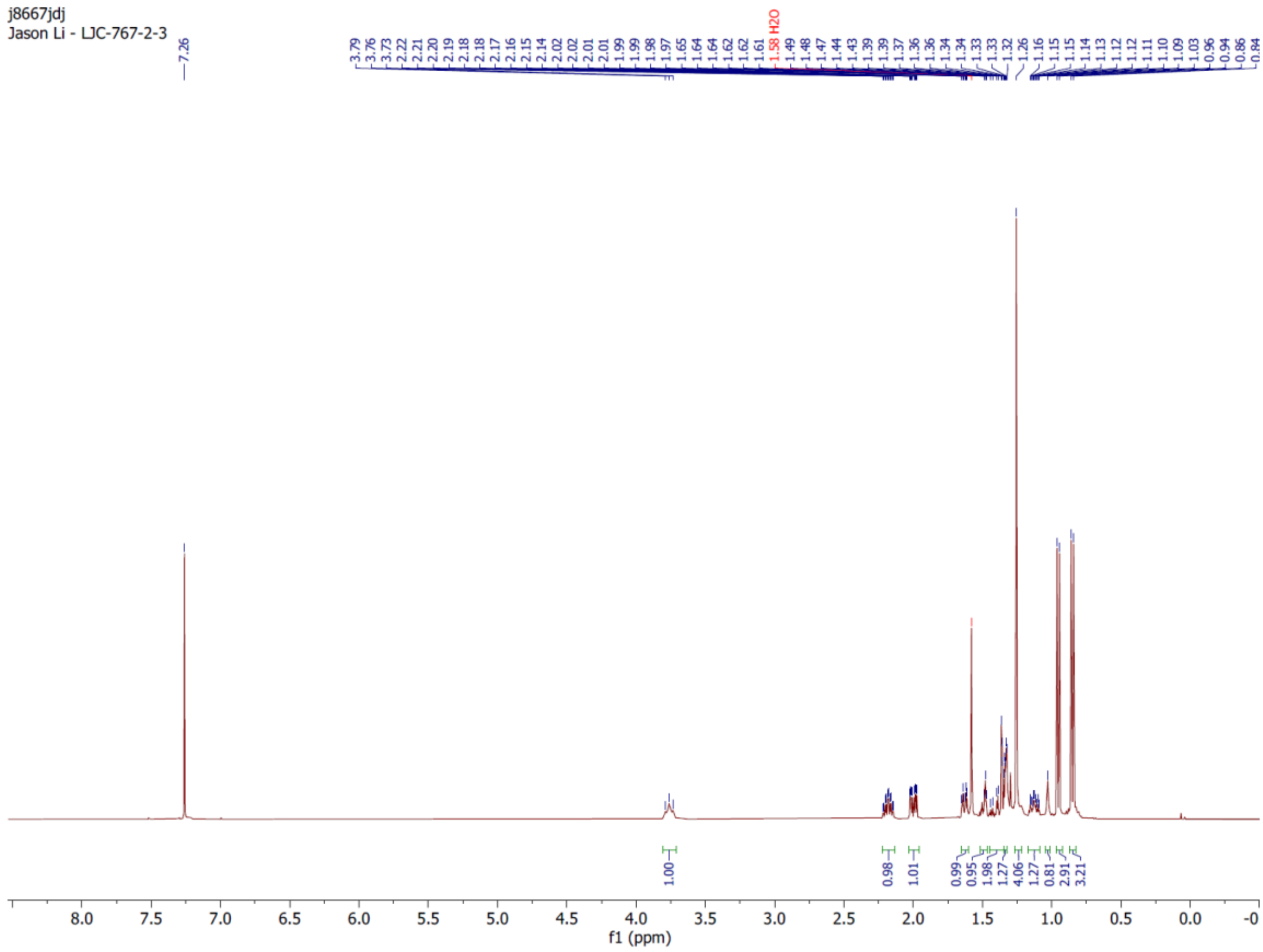

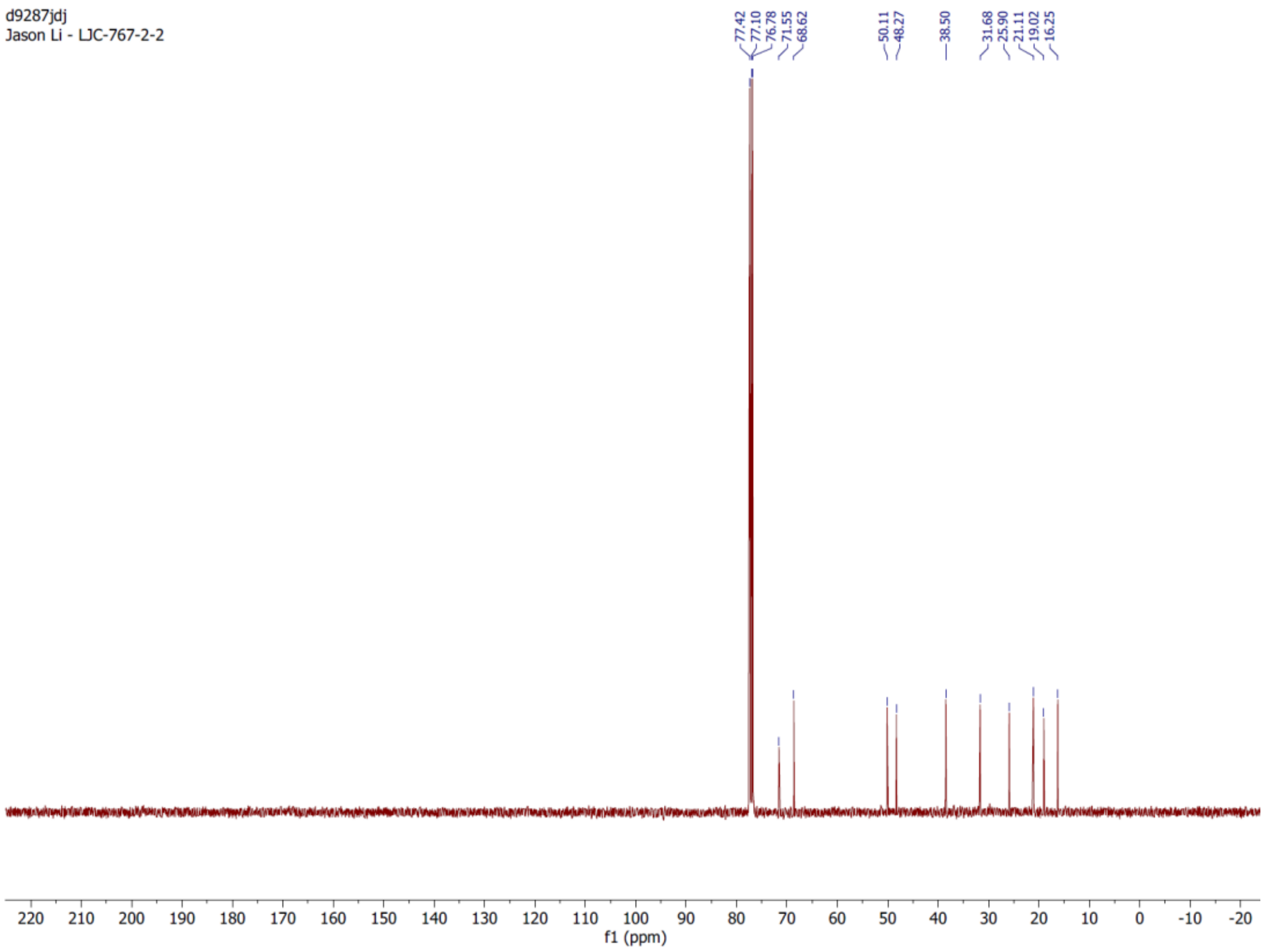

8. Modelling using Autodock VINA

Automated docking was performed using AUTODOCK VINA 1.1.2.[9] Coordinates for the (-)-menthol **8** were prepared using ELBOW[10] in PHENIX.[11] The appropriate pdbqt files for the models *Dch*UPO, artUPO and the ligand were prepared in AUTODOCK Tools from the structure. Heme with Compound I was modelled into the structures using P450 structure 1DZ9.[12] The active site of *Dch*UPO was contained in a grid size of 36 Å × 36 Å × 36 Å (corresponding to x, y, z) with 1 Å spacing, centred around the catalytic centre at positions -19.55 Å × -1.13 Å × 8.38 Å (corresponding to x, y, z). The active site of artUPO (7ZNV[13]) was contained in a grid size of 34 Å × 34 Å × 34 Å (corresponding to x, y, z) with 1 Å spacing, centred around the catalytic centre at positions -2.55 Å × -5.28 Å × -27.21 Å (corresponding to x, y, z). These values were generated using AutoGrid in the AUTODOCK Tools interface. The dockings were performed by VINA, therefore the posed dockings were below 2 Å r.m.s.d. The results generated by VINA were visualised in AUTODOCK Tools 1.5.6 where the ligand conformations were assessed based upon lowest VINA energy. **Figure 2** shows the active sites of *Dch*UPO with (-)-menthol **8** superimposed with the active site of artUPO with the same substrate.

9. References

1. W. Kabsch, *Acta Crystallogr. D Biol. Crystallogr.*, 2010, **66**, 125-132.

2. P. Evans, *Acta Crystallogr. D Biol. Crystallogr.*, 2006, **62**, 72-82.

3. G. Winter, *J. Appl. Crystallogr.*, 2010, **43**, 186-190.

4. A. Vagin and A. Teplyakov, *J. Appl. Crystallogr.*, 1997, **30**, 1022-1025.

5. J. Jumper, R. Evans, A. Pritzel, T. Green, M. Figurnov, O. Ronneberger, K. Tunyasuvunakool, R. Bates, A. Žídek, A. Potapenko, A. Bridgland, C. Meyer, S. A. A. Kohl, A. J. Ballard, A. Cowie, B. Romera-Paredes, S. Nikolov, R. Jain, J. Adler, T. Back, S. Petersen, D. Reiman, E. Clancy, M. Zielinski, M. Steinegger, M. Pacholska, T. Berghammer, S. Bodenstein, D. Silver, O. Vinyals, A. W. Senior, K. Kavukcuoglu, P. Kohli and D. Hassabis, *Nature*, 2021, **596**, 583-589.

6. P. Emsley and K. Cowtan, *Acta Crystallogr. D Biol. Crystallogr.*, 2004, **60**, 2126-2132.

7. G. N. Murshudov, A. A. Vagin and E. J. Dodson, *Acta Crystallogr. D Biol. Crystallogr.*, 1997, **53**, 240-255.

8. B. Melling, T. Mielke, A. C. Whitwood, T. J. C. O'Riordan, N. Mulholland, J. Cartwright, W. P. Unsworth and G. Grogan, *Chem Catal.*, 2024, **4**, 100889.

9. O. Trott and A. J. Olson, *J. Comput. Chem.*, 2010, **31**, 455-461.

10. N. W. Moriarty, R. W. Grosse-Kunstleve and P. D. Adams, *Acta Crystallogr. D Biol. Crystallogr.*, 2009, **65**, 1074-1080.

11. P. D. Adams, P. V. Afonine, G. Bunkóczi, V. B. Chen, I. W. Davis, N. Echols, J. J. Headd, L. W. Hung, G. J. Kapral, R. W. Grosse-Kunstleve, A. J. McCoy, N. W. Moriarty, R. Oeffner, R. J. Read, D. C. Richardson, J. S. Richardson, T. C. Terwilliger and P. H. Zwart, *Acta Crystallogr. D Biol. Crystallogr.*, 2010, **66**, 213-221.

12. I. Schlichting, J. Berendzen, K. Chu, A. M. Stock, S. A. Maves, D. E. Benson, R. M. Sweet, D. Ringe, G. A. Petsko and S. G. Sligar, *Science*, 2000, **287**, 1615-1622.

13. W. X. Q. Robinson, T. Mielke, B. Melling, A. Cuetos, A. Parkin, W. P. Unsworth, J. Cartwright and G. Grogan, *Chembiochem*, 2023, **24**, e202200558.
